# Development of the N400 for Word Learning in the First 2 Years of Life: A Systematic Review

**DOI:** 10.3389/fpsyg.2021.689534

**Published:** 2021-06-30

**Authors:** Caroline Junge, Marlijne Boumeester, Debra L. Mills, Mariella Paul, Samuel H. Cosper

**Affiliations:** ^1^Department of Experimental Psychology, Utrecht University, Utrecht, Netherlands; ^2^School of Psychology, Bangor University, Bangor, United Kingdom; ^3^Psychology of Language Research Group, Georg-August-Universität Göttingen, Göttingen, Germany; ^4^Institute of Cognitive Science, University of Osnabrück, Osnabrück, Germany

**Keywords:** infants (birth to 2 years), N400, event related potentials, lexicon acquisition, word learning, semantic processing, language acquisition

## Abstract

The N400 ERP component is a direct neural index of word meaning. Studies show that the N400 component is already present in early infancy, albeit often delayed. Many researchers capitalize on this finding, using the N400 component to better understand how early language acquisition unfolds. However, variability in how researchers quantify the N400 makes it difficult to set clear predictions or build theory. Not much is known about how the N400 component develops in the first 2 years of life in terms of its latency and topographical distributions, nor do we know how task parameters affect its appearance. In the current paper we carry out a systematic review, comparing over 30 studies that report the N400 component as a proxy of semantic processing elicited in infants between 0 and 24 months old who listened to linguistic stimuli. Our main finding is that there is large heterogeneity across semantic-priming studies in reported characteristics of the N400, both with respect to latency and to distributions. With age, the onset of the N400 insignificantly decreases, while its offset slightly increases. We also examined whether the N400 appears different for recently-acquired novel words vs. existing words: both situations reveal heterogeneity across studies. Finally, we inspected whether the N400 was modulated differently with studies using a between-subject design. In infants with more proficient language skills the N400 was more often present or showed itself here with earlier latency, compared to their peers; but no consistent patterns were observed for distribution characteristics of the N400. One limitation of the current review is that we compared studies that widely differed in choice of EEG recordings, pre-processing steps and quantification of the N400, all of which could affect the characteristics of the infant N400. The field is still missing research that systematically tests development of the N400 using the same paradigm across infancy.

## Introduction

One remarkable feat of infancy is that infants start comprehending words from a complex environment full of objects and sounds (Quine, 1960; Smith and Yu, 2008). There is behavioral experimental evidence that at least by 6 months, typically-developing infants can match some concrete words such as “mommy” or “sock” to their correct referents (Bergelson and Swingley, 2012; Tincoff and Jusczyk, 2012). This line of research used infant eye or head movements to assess early comprehension (see also Swingley, 2005). For instance, in cross-modal preferential looking paradigms infants see multiple objects while hearing a word that matches only one of the objects presented. Research showed that upon hearing this target word, infants fixated the object that matched the spoken word significantly more often and faster than objects that did not match the spoken word (Golinkoff et al., 2013), an effect that further increased with age (Fernald et al., 1998). Other researchers have used this paradigm to demonstrate evidence of semantic priming, a process which reflects that a prior given context can shape a child's expectations of the upcoming words and thereby reduce semantic integration efforts. For example, 24-month-olds looked longer at the requested object when the target word (e.g., “bike”) was preceded by a semantically-related word (e.g., “lorry;” Arias-Trejo and Plunkett, 2013). There are also other means to test a child's word comprehension. Some researchers have inferred word comprehension based on whether or not infants reached or pointed to a correct referent out of a multi-object display (e.g., Woodward et al., 1994). Besides experimental evidence, parental reports such as the widely-available MacArthur Communicative Development Inventory (Fenson et al., 1994; Frank et al., 2017b) have also proven informative and have further underscored that for most infants, first signs of early word comprehension had started at least by 8 months (currently the youngest age sample).

Crucially, differences in early vocabulary sizes might already differentiate typical from atypical language development. Research documented that various atypical populations often have smaller vocabularies compared to their typically-developing peers, such as is the case with infants at elevated risk of developing autism spectrum disorder (Ference and Curtin, 2015; Lazenby et al., 2016), those at risk of developmental dyslexia (van Viersen et al., 2017), or late talkers (Fernald and Marchman, 2012). This makes it vital to understand how infants start their vocabulary acquisition, and which factors contribute to variation across infants. Fortunately, there is a considerable body of research correlating potential factors with children's vocabularies (such as variation in maternal speech input; for a recent review see Kidd and Donnelly, 2020). Researchers also turn to carefully designed experiments to assess potential influences, which allows for a level of control of the environment that cannot be achieved with naturalistically-obtained measures. However, demonstrating that infants understand some words *via* behavioral research is not a straight-forward task, as infants do not always show the desired behaviors and keep on task. As a result, drop-out rates in infant studies are typically high (Bergmann et al., 2018) and null-effects based on infant preferences remain difficult to interpret. That is, from an absence of looking preference or correct pointing one cannot infer infants do not recognize the requested items; it could be that the distracter items were equally or more attractive (Pruden et al., 2006; Aslin, 2007; Junge et al., 2020).

Another way to investigate infant vocabulary in laboratory settings is by recording their electroencephalogram (EEG) while infants' brains process words. An advantage of this technique is that it provides without any overt response from the child an on-line measure of language processing with high temporal resolution, allowing to directly and precisely measure when different computational processes underlying language comprehension are taking place in the brain. This method has proven increasingly popular for testing infants with the advance of neuroimaging techniques (Reid, 2012; Azhari et al., 2020). There is increasing literature explaining and improving the methodology for testing such young populations (e.g., Thierry, 2005; De Haan, 2007; Bell and Cuevas, 2012; Stets et al., 2012; van der Velde and Junge, 2020). One of the most commonly derived measures from the EEG is the Event-Related Potential (ERP) technique. In ERP paradigms, participants are presented with certain types of stimuli multiple times while their EEG is continuously being recorded. The ERP represents the averaged brain activity patterns to one type of stimuli within a short time window, beginning at the onset of a stimulus (“time locked”). Researchers then compare ERPs time-locked to different kinds of stimuli types as a proxy for changes in behavior to ascertain whether and when infants can discriminate between these stimuli types, such as familiar words vs. novel words (either phonotactically legal words called pseudowords or impossible words called non-words), or congruous vs. incongruent object-word pairings. To illustrate the latter, infants might see a picture of a common object (e.g., a shoe), and then hear a word that either matches (e.g., “shoe”) or does not match the picture context (e.g., “car”). A consistent difference in ERP amplitudes between the two conditions from word onset then signals that infants' brains process words differently depending on the goodness of fit with the semantic context, and thus reflects that infants are sensitive to object-word pairings.

Research on word processing in adults has repeatedly identified one component associated with lexical-semantic processing: the N400. Compared to a congruent condition where words and object referents are presented correctly with one another, words that are not or less plausible in a meaningful context elicit an ERP component that has a more negative amplitude, peaking around 400 ms after the onset of the word, with a broad scalp distribution most clearly visible on centro-parietal electrodes (relative to mastoid sites). This component was first described in Kutas and Hillyard (1980), and has since then been elicited in over a 1,000 studies (for an overview, see Kutas and Federmeier, 2011). These three characteristics (polarity, latency, and scalp distribution) together define the physical characteristics of the N400, and allow us to make comparisons across studies. The N400 is elicited by a range of “meaningful contexts:” ranging from various sentence contexts (Rabovsky et al., 2018), to single words (e.g., Rugg and Nagy, 1987), to word repetitions (e.g., Rugg et al., 1995), and to a picture or other visual context (e.g., Barrett and Rugg, 1990). While its latency (that is, time window chosen for analysis) is remarkably constant as it falls between 200 and 600 ms post word-onset, in adults it is the amplitude that usually shows sensitivity to a range of semantic manipulations (Kutas and Federmeier, 2011).

Researchers vary in their interpretation of the adult N400, particularly on its functionality. Historically, two main theories have prevailed: spreading activation (cf. Posner and Snyder, 1975) and semantic integration (for reviews, see Lau et al., 2008; Kutas and Federmeier, 2011). Spreading activation describes an automatic process in which activation is then forwarded from a prime to an associated item, whereas semantic integration is a process in which prime and target are related for a combined meaning. Within semantic integration, some view the N400 to reflect processes associated with post-lexical integration of words into the given context (Hagoort et al., 2009; Baggio and Hagoort, 2011), whereas others position the component to be at the level of semantic access (Van Berkum, 2009; Thornhill and Van Petten, 2012). Nevertheless, whatever its interpretation is, it is clear that the N400 in adults indexes a broad sensitivity to lexical-semantic processing ranging from lower-level to higher contextual factors, indicating that the presence of the N400 can reflect both automatic and more controlled lexical semantic processes (Bentin, 1987; Holcomb, 1988; Kiefer, 2002; Lau et al., 2008). This makes the N400 component an ideal component to study the emerging vocabularies in infancy and factors that contribute to word learning.

The first researchers to observe an N400-like effect (that is, with a negative shift) in infants were Friedrich and Friederici (2004). In this study, incongruous picture-word pairs elicited a more negative wave than congruous picture-word pairs in German 19-month-olds. The effect in these infants differed from adults in both time window and topography. That is, the effect occurred later, between 800 and 1,400 ms after word onset, and was more broadly distributed across the brain (compared to a centro-parietal distribution in adults). Nevertheless, the N400 effect in infants suggests that they already have the mechanisms needed to integrate word meaning into a semantic context. Around the same time, Mills and colleagues summarized in a chapter evidence with American-English 13- and 20-month-old infants an N400 effect, albeit with a similar timing and duration to that typically observed in adults (Mills et al., 2005a).

The earliest age at which an N400 effect has been found with lexical-semantic violations is at 6 months (Friedrich and Friederici, 2011). In this study, infants learned associations between pseudowords and new objects during a training phase. Pseudowords were either constantly preceded by the same novel object, enabling the learning of object-word mappings, or by a different novel object during each presentation, making it impossible to learn object-word mappings. After five presentations, ERPs showed a reduced negativity for the words in the constant pairing condition compared to the words in the rotated pairing condition between 600 and 900 ms after word onset, indicating the encoding of new object-word mappings. This N400 priming effect was found in parietal regions. However, during the test phase 1 day after training, the infant-ERPs did not show a semantic priming effect when the learned words were preceded by congruent or incongruent objects. In studies with a similar design, the N400 effect found during training in infants aged 6 months was not replicated in younger infants of 3 months old (Friedrich and Friederici, 2017), nor was it observed in infants of 6–8 months of age (Friedrich et al., 2017). In the latter study, infants either took a nap or stayed awake for about an hour between the training and test phase. During the test phase, they were presented with congruent and incongruent picture-word pairs. Only the infants who took a relatively long nap showed a semantic priming effect despite not showing evidence of encoding at the end of the training phase. Together, these studies suggest that around 6 months, infants start having the neural resources to quickly encode new object-word mappings but are not able yet to build strong associations between words and meanings in long term semantic memory. Studies with older infants show that from the age of at least 14 months infants are able to consolidate newly acquired lexical-semantic knowledge into long term memory, reflected by N400 priming effects 1 h after training independently of whether infants took a nap (Friedrich et al., 2019, 2020), and after 1 or 2 days after training (Friedrich and Friederici, 2008).

However, in contrast to adult studies, there is no clear consensus on how to examine the N400 and to select characteristics for statistical analyses such as time window and scalp distribution. Most infant studies reporting N400 rely on *post-hoc* visual inspection of the grand-averaged ERPs or on bottom-up non-parametric statistical analyses to identify time windows in which conditions maximally differ. As a result, infant studies widely differ in how they quantify the N400 component of interest, even though they all consider their component of interest to reflect the N400. To illustrate, some use a time window as early as 200–600 ms (Sheehan et al., 2007; Friedrich and Friederici, 2008), whereas others use a later time window from 400 to 500 ms onwards (Junge et al., 2012a; Borgström et al., 2015a), or even later (Friedrich and Friederici, 2004; Torkildsen et al., 2008). Recall that in adults, the latency of the N400 proved rather constant between 200 and 600 ms. While some infant studies hence show a delay in timing, others suggest that the N400 appears remarkably similar in infants compared to adults.

This lack of consensus further obscures comparison across studies and limits our observations whether there is development in the N400 from infancy to adulthood. Understanding whether there is development is necessary to advance our theory-building. Some theorize for instance that semantic processing abilities emerge prior to the onset of and are therefore considered independent of grammatical and syntactic processing skills (e.g., Morgan et al., 2020). Moreover, it prevents the field from making clear predictions on how to analyze the infant N400. This would benefit future studies that would like to use pre-registrations or registered reports in which analysis plans need to be thoroughly described prior to data collection (as suggested in Frank et al., 2017a; Paul et al., 2021).

There is ample reason to believe that the infant N400 is delayed compared to that observed in adults. A cross-sectional study with children between 5 and 23 years shows that its peak latency is far from adult-like: it decreases with age and only becomes stable by 13 years of age (Holcomb et al., 1992). Moreover, similar comparisons on latency of other components also reveal delays in development, even though they appear both functionally and physically somewhat similar to its adult counterpart. For instance, early auditory components such as the N1-P2 complex, which is a robust and automatic response to auditory stimuli, only becomes adult-like by mid-puberty (Pasman et al., 1999). The mismatch negativity response, which in adults is a negative peak to deviant sounds peaking around 100–250 ms (Näätänen, 1990), can occur in infants not only as a negativity but also as a positivity, and usually longer-lasting (e.g., Leppänen et al., 2004). Finally, the N170, which in adults is an early-negative component sensitive to the presentation of human faces (Bentin, 1987; Eimer, 2011), is delayed by another 100–150 ms for infants (therefore called the “N290;” Halit et al., 2003). Together, developmental studies on different sorts of cognitive processing all seem to suggest that while infants display distinct ERP components similar to adults, it is not fully matured.

There are several explanations why infant ERPs appear different from adult ERPs. One obvious explanation is that infant brains are still developing compared to that of adults, and such changes are clearly visible in the EEG (see also DeBoer et al., 2007). Compared to adults, infant EEG has more slow-wave activity (Taylor and Baldeweg, 2002), with noticeable larger amplitudes. The infant ERP does not resemble the typical adult-like peaked responses, but reveals slower and more global components. There are also changes in spatial and temporal distribution, with infant responses usually appearing as more “smeared” responses and are considered less well-defined than in adulthood (De Haan, 2007). These differences in the EEG rhythm and in the ERP waveform morphology are presumably reflecting infant developmental changes in maturational brain processes such as synaptic density and myelination. Infant skulls are also thinner and their fontanels are still in the process of gradually closing, both of which impacts the amplitude and latency with which EEG is recorded on the skull (DeBoer et al., 2007; De Haan, 2007).

Besides changes in brain maturation, infancy marks great development in the cognitive processes that contribute to the presence of ERP components. This period marks great advances in vocabulary achievements. Although semantic processing only becomes evident in infants from 6 months on, when words are slowly added to their vocabulary, there appears acceleration in their second year of life, a phenomenon called the vocabulary spurt (Goldfield and Reznick, 1990). Despite the debate whether this transition from the acquisition of slowly learned words to faster rates of word acquisition resembles two distinct modes of word learning (Nazzi and Bertoncini, 2003), it appears that older infants process words faster than younger infants (Fernald et al., 1998, 2006; Bergelson and Swingley, 2012). Not only does the rate of word acquisition change in the second year of life, but also supportive cognitive systems, such as increasing memory abilities (Gershkoff-Stowe, 2002) and increased visual and auditory acuity, show great progress (Werker and Hensch, 2015). With age, children also have more experience with processing sounds and visual stimuli, and thus more exposure to different speakers producing different words in different contexts, thus bolstering their word learning skills. Moreover, changes in infants' alertness state also impacts the elicitation of language-related ERP components (Friedrich et al., 2004). We therefore ask whether there is any development visible in the N400 when zooming in on the first 2 years of life.

To investigate whether there is development, the current review systematically compared studies on the physical characteristics of the N400 elicited in infants who listened to words, thereby pooling all available evidence together. We asked how the infant N400 is quantified in terms of latency and distribution in order to examine whether there is any development in how the N400 develops in the first 2 years of life. Our ultimate goal is to develop a better understanding of what characterizes the N400 in infancy. Note that we could not conduct a meta-analysis, since it was precisely how each study quantified the dependent variable (N400) rather than the results itself that proved informative to our research question.

While we focussed on those situations that involved lexical processing and as a consequence of this, elicited the N400, it is noteworthy that there are also situations that elicited the N400 in infants that did not involve linguistic processing. For instance, action-perception paradigms, in which expectancies of meaningful actions can be violated, often report an infant N400. To illustrate, infants might see a person with a spoon, but this person brings the spoon either to her mouth (expected action) or to her forehead (unexpected action). Reid and colleagues show that infants as young as 5 months already elicited an N400 (Reid et al., 2009; Kaduk et al., 2016; Michel et al., 2017). Action perception and linguistic processing have similar underlying neural mechanisms and brain structures in adults (Iacoboni, 2005) and are thus reflected by comparable N400 components. However, these effects differ in young infants. In action-perception literature, the infant N400-like component is only detectable in the unexpected condition, visible as a peak with absolute negative amplitude, but this peak is absent in the expected condition, which is instead characterized by a large negative component associated with attention: the Negative Central component (Reid et al., 2009). This contrasts with the N400 indexing word learning, which becomes visible as a negative peak for the incongruent condition relative to the congruent condition. Thus, the maturation of the action N400 effect may develop on a different trajectory than the linguistic N400 effect for word learning (cf. Michel et al., 2017). We therefore did not include such action-perception literature on the infant N400 in our systematic review.

For linguistic processing, few studies directly compared the N400 using the same lexical-priming paradigm at different age ranges across infancy. One of the first were Friedrich and Friederici (2004, 2005a), who tested 12-, 14-, and 19-month-olds cross-sectionally. They showed that while 14-month-olds resembled the 19-month-olds and showed a late N400, the 12-month-olds did not. The 12-month-olds displayed only a N200–500, a component associated with word form familiarity (Kooijman et al., 2005), possibly reflecting phonological priming of the upcoming word form. Crucially, the youngest age group did not display the N400. Similarly, Rämä et al. (2013), who compared infants of 18 and 24 months old, observed an N400 priming effect in the older age group, which was absent in the younger age group. Borgström et al. (2015b) carried out a longitudinal study with 20- and 24-month-olds. Their results also suggest that the N400 becomes mature-like with age. While as 20-month-olds the infants had a relatively late N400, and only for known words, at 24 months the same children had an earlier N400 that was present for both known words as well as for novel words whose meanings were acquired in the same visit. These studies suggest that time window and distribution of the N400 are possibly modulated by age in infants. That is, whereas the N400 effect had a relatively late onset (i.e., 400 ms after word onset) in the group of 19-month-olds (Friedrich and Friederici, 2004), the 24-month-olds in the study by Rämä et al. (2013) and Borgström et al. (2015b) showed an earlier N400 onset, more similar to adults.

There are also comparable shifts in its distribution characteristics: the N400 effect in the 19-month-olds had a broad topographical distribution comprising frontal, central, parietal, and temporal areas, whereas the N400 effect in the 24-month-olds in the study by Rämä et al. (2013) had a more adult-like distribution focused on parietal electrodes. Therefore, we hypothesized that with age, the N400 would appear more adult-like, both in terms of timing and in a more focal, centro-parietal distribution.

While we reason that there is development within the first 2 years of life, it is also possible that infancy (between 6 and 24 months of age) is too small a period to note substantial differences in the physical characteristics of the linguistic N400. In line with this null-hypothesis there is another ERP study with a cross-sectional semantic-priming design which reported no differences between 13- and 20-month-olds, as long as the children understood all of the words and saw all of the pictures included in the analyses (Mills et al., 2005a). This null-hypothesis also concurs with other studies on different components. For instance, it appears that there is little change with ERP components specific to face-categorization (Di Lorenzo et al., 2020).

Moreover, age cannot be the only factor contributing to variety in N400 results in infants. That is, although Friedrich and Friederici (2005b) did not find an N400 effect in all 12-month-olds (cf. Friedrich and Friederici, 2006), other studies found an N400 effect in even younger infants (e.g., Friedrich and Friederici, 2011; Junge et al., 2012a). It is likely that experimental designs also impacted the presence of the N400. While the N400 has been shown to appear both in situations testing knowledge of familiar words (pre-existing object-word relationships), others tested novel words by adding a training phase in which novel words were linked to novel objects. We hypothesized that recognizing incongruities for familiar words would be easier than words learned recently within the same lab visit. Thus, an effect of experimental design would reveal itself as novel-word learning paradigms resulting in an N400 being more delayed, and/or more broadly distributed compared to simpler paradigms relying on pre-existing knowledge.

Finally, we examined whether certain characteristics of the N400 effect could be linked to subgroup characteristics, such as later language outcomes. Some studies examined whether subgroups of infants differ in their realization of the N400 as a function of individual language profiles. For instance, while 12-month-olds in general did not show evidence of the N400 in a picture-priming paradigm (Friedrich and Friederici, 2005b), a re-analysis that took into account current vocabulary size showed that some infants—those with a relatively large expressive vocabulary—actually elicited the N400 (Friedrich and Friederici, 2010). We reasoned that infants with relatively larger vocabularies might find the same task easier than infants with smaller vocabularies. In such cases, the N400 might be more mature and more focally-distributed, compared to their age-matched peers, for whom the N400 would be delayed and more widely distributed, or even absent.

To summarize, this study aims to learn more about how characteristics of the infant N400 are modulated by age; methodological parameters, such as real words vs. novel words; and individual differences, such as later language outcomes. We reasoned that with increasing age, with real words, and in infants with relatively good language skills, the N400 would appear more adult-like than in other testing situations. That is, with increasing age, with increasing vocabularies, and in cognitively less-taxing paradigms, we hypothesized that the linguistic infant N400-like component will manifest itself with an earlier latency and predominantly restricted to centro-parietal distributions. Our aim is that this systematic review contributes to a better understanding of the N400 in infants and functions as a guidance for future researchers in determining their time windows and regions of interest when studying the N400 in word learning.

## Methods

The current systematic review followed the Preferred Reporting Items for Systematic Review and Meta-Analysis guidelines (PRISMA, Moher et al., 2009).

### Data Source and Search Strategy

Electronic databases PubMed, Google Scholar, and Web of Science were systematically searched for published articles on April 28th 2020, and checked for updates on January 7th 2021. Search strategies included terms corresponding to three broad components: semantic processing, infants, and electrophysiological methodology. Search strings were adapted for each electronic database. For instance, for Web of Science we used: “semantic” OR “word” OR “lexic^*^” OR “senten^*^” OR “vocabulary” OR “speech” AND “infan^*^” OR “toddler^*^” OR “child” OR “children” AND “EEG” OR “ERP” OR “N400” OR “event-related potential^*^”. Full search strategies for each database can be found in Appendix A. Searches were restricted to studies published in English. Finally, a forward and backward citation search was performed in order to find any studies not identified through electronic search. Then the authors screened titles, abstracts, and full texts to find eligible studies.

### Data Selection of Eligible Studies: Inclusion and Exclusion Criteria

Publications describing original research were included in the systematic review if they (1) were written in an English, peer-reviewed journal; (2) reported results on typically developing infants between 0 and 24 months old, either as test or control group; (3) reported EEG/MEG studies using tasks relevant to elicit an N400 component or effect; and (4) used linguistic stimuli to study semantic processing. Publications were excluded if (1) N400 results of the same experiment were also reported in a previous publication, (2) children were older than 24 months, (3) studies did not test semantic processing, (4) they did not report an N400 component with a negative polarity, or (5) they appeared as conference proceedings. Furthermore, we excluded reviews, systematic reviews, pre-prints, and meta-analyses.

Reference management program RefWorks was used to store retrieved publications. Titles and abstracts were screened to identify potentially relevant publications. Eligibility of these publications was then assessed by reading the full text and by taking into account the previously stated eligibility criteria. The selection process was carried out by the second author and checked by the fourth and final authors. All authors discussed cases of doubt.

### Data Extraction

For each selected publication that fit our eligibility criteria, we noted information about the (1) publication (authors, publication year, journal); (2) design; (3) analysis; and (4) results. With respect to its design, we extracted information about: sample characteristics (e.g., mean age; final sample size; additional participant groups), and paradigm characteristics (e.g., choice of paradigm; stimuli context which refers to the prime-target structure; word type categorized as familiar “existing” words, as pseudowords, or as non-words). For the ERP measurement characteristics we noted down: whether the analysis was based on visual inspection of data, on a data-driven approach using for example fixed time bins or clustered permutation tests, or predefined from previous literature; choice of reference for offline analysis; onset and offset for time windows; and distribution characteristics. For those studies that statistically tested consecutive bins of 100 or 200 ms time windows rather than testing one larger time window we reported the first time window in which an increased negativity was reported for the incongruous condition vs. congruous condition, and merged this with consecutive time windows in which the effect persisted. For instance (Torkildsen et al., 2008) reported congruity effects for real words for time windows 400–600, 600–800, but only a local effect for the 200–400 ms time windows; here, we selected 400–800 ms. For distribution we made two columns: one on the anterior-posterior axis (frontal, central, posterior), and one the laterality-axis (left hemisphere, middle, right hemipshere). Distribution effects were only noted as such when the omnibus ANOVAs pointed to interactions between Condition x Distribution; otherwise they were considered broad. Whenever there were multiple conditions, we relied whenever possible on omnibus ANOVAs that captured main effects of or interactions with condition.

### Data Analysis

Since our paper takes a component-centered approach we only focus on studies that explicitly listed the N400 effect with a negative-going polarity. To examine whether N400 characteristics are modulated by age, experimental designs, or individual differences, for the remainder of our analyses we decided to focus on studies with the semantic-priming paradigm, as the large majority of reported studies use this paradigm (see Result section below).

To explore whether the choice of time window of the N400 was affected by participant age, we provided scatterplots of the records depicting the onset latency, offset latency, and time interval by age. We used simple correlation tests to index their significance. Next, we examined the relationship between topographical distributions and age. For the distributions, we provided pie charts on three different age groups: 6–11-month-olds; 13–18-month-olds; and 19–24-month-olds. We examined the axes of distribution separately, the anterior-posterior axis and the laterality-axis (depicting hemispheric differences). To explore age group differences, we carried out Chi-square tests with Age group (3), and focal or broad distributions coded as categorical variables.

To examine variations with experimental paradigms we grouped records based on whether they only concerned existing words or also novel words. We then compared latency and distribution factors using Independent-Samples *T*-tests.

Finally, to explore the relationship between physical properties of the linguistic N400 effect and other individual characteristics besides age we examined records that used a within-subject, between-subject-design or a combination of the two. Here, we split records depending on the type of contrast made in their designs (between- vs. within-subjects). To illustrate, some within-subject studies provide additional information about a child's current vocabulary development, whereas other within-subject studies examine elicitations of the N400 in the same children in multiple contexts, such as known words as well as novel words. Between-subject studies, in contrast, test elicitation of the N400 effect across different groups of children, who differ for instance in language background or in experiential designs. We only provide descriptive statistics, as the comparisons for within-subject and between-subject studies may both yield in some case categorical differences (e.g., a presence of the N400 effect in one situation, coupled with an absence in another situation), continuous differences (e.g., a delayed N400 in one situation compared to another), or no apparent differences.

## Results

### Literature Search

**Figure 1:**
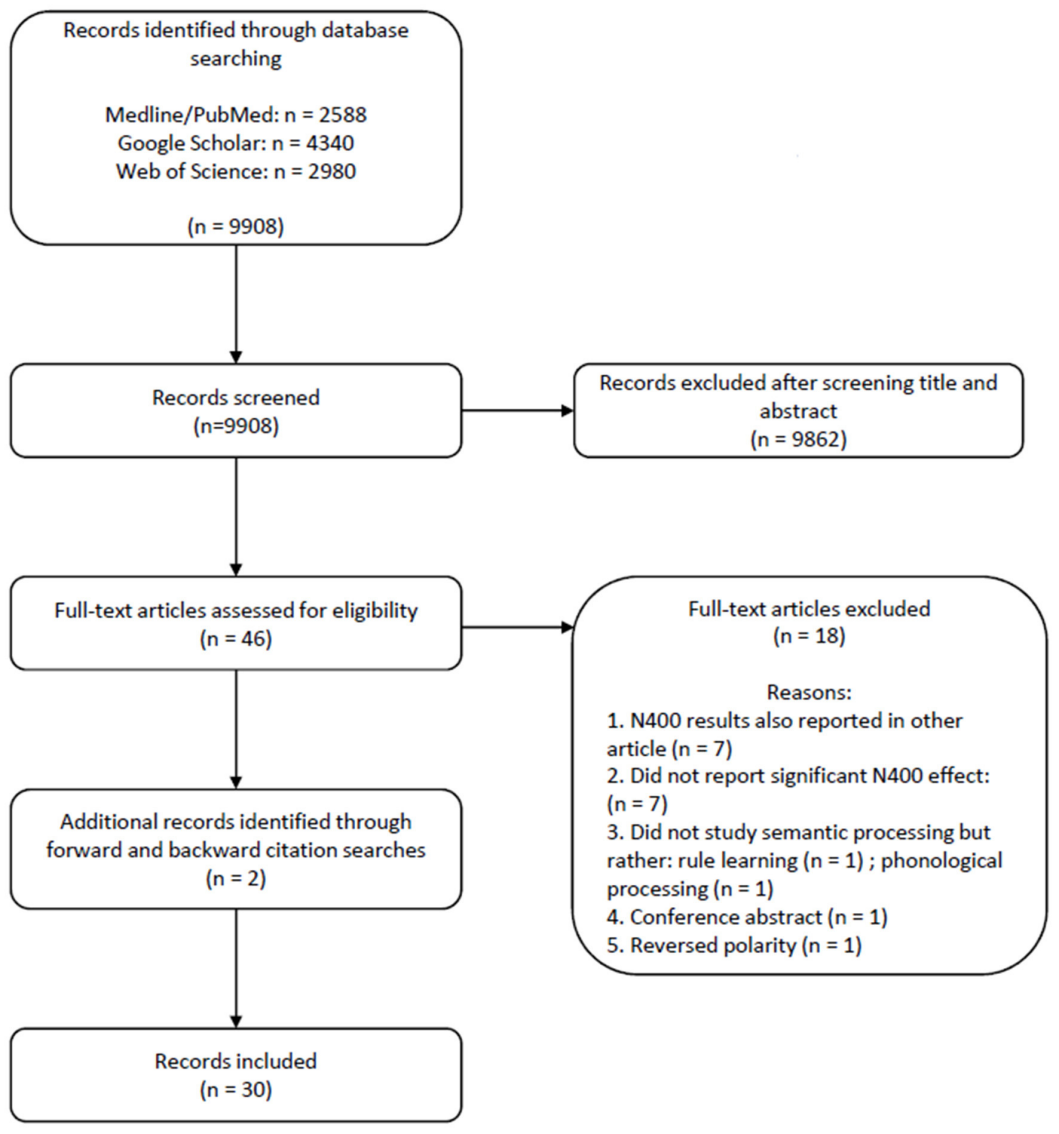
PRISMA flow diagram of the systematic literature search process (Moher et al., 2009).

Figure 1 provides the PRISMA flow chart of our systematic literature search. The search strategy yielded 9,908 results: 2,588 from PubMed (Medline), 4,340 from Google Scholar and 2,980 from Web of Science. Of these, 9,862 were excluded based on title and abstract, and an additional 14 records were excluded after reading the full text. Reasons for exclusion included N400 results that were also reported in a previous publication and methodologies that were not designed to study semantic processing but rather rule learning and phonological or phonotactic processing. We removed publications reporting N400 with a positive polarity (*n* = 1), or those that only reported on other components, such as the N200-500 (*n* = 6), and we removed two samples with null-findings. Two additional records were identified *via* forward and backward citation search.

Some records tested multiple samples, in which case we listed each sample separately. Whenever there were multiple conditions while studies tested the same sample of infants within the same recording session, we relied on omnibus ANOVAs to report main effects of condition (e.g., studies testing both pseudowords and novel words); otherwise, we listed the conditions separately (which was the case in one record). Finally, 32 articles met all inclusion criteria and were included in the present review. Two of these studies followed up earlier described samples as they contained additional information such as subsequently collected information on language development. In these cases, we merged the data on additional subject characteristics with the original data file. Thus, we kept 30 publications (“records”), which yielded 35 infant samples (“samples”), which in total provided 36 unique statistical analyses (“analyses”) with the N400 infant-like as a dependent variable indexing linguistic processing^1^.

### Study Characteristics

Table 1 provides characteristics of the 35 included samples from the 30 publications that reported N400-component as a proxy of lexical-semantic processing in infants. For clarity reasons, each row reports results of the full sample of included infants from that publication unless stated otherwise (i.e., results of subgroups of high and low vocabulary size are instead reported in Table 2). All samples included monolingual infants only. When a publication included multiple age groups, we presented the results of each age group separately. All but one publication recorded EEG; the other MEG. Studies are in alphabetical order sorted by first author of the original publication. All publications were peer-reviewed and published between 2004 and January 2021. Most publications included one sample of infants (*n* = 25/30); others included multiple age-samples. Some studies tested the same infants twice, with a fixed interval (*n* = 2/30): here the second sample yielded usually smaller sample sizes. In total 1,114 infants contributed data. The number of included infants in samples ranged from 12 to 107 (mean = 31.8, SD = 20.3; median = 28). Age of the included infants ranged from 6 to 24 months old (mean = 16.8 months; SD = 5.1 months; median 18 months).

**Table 1:** List of papers from the literature search that yielded N400 results.

**Records**	**References**		**Age (months)**	**Sample Size**	**Sub-groups**	**Paradigm**	**Stimuli context**	**Word type**	**Learning phase**	**N400 selection**	**Reference electrode(s)**	**N400 TW onset**	**N400 TW offset**	**Distribution:** **ant-post**	**Distribution:** **laterality**
1	Asano et al. (2015)		11	19	No	Match/mismatch	Picture-word priming paradigm	Pseudowords	No	P	M	350	550	C	LL/M/RL
2	Borgström et al. (2015a)	EXP1	20	38	Yes; vocabulary	Match/mismatch	Picture-word priming paradigm	Existing words	Yes	P	A	500	900	B^*^	LL/M/RL^*^
		EXP2	24	34	Yes; vocabulary	Match/mismatch	Picture-word priming paradigm	Existing words	Yes	P	A	500	900	B^*^	LL/M/RL^*^
3	Borgström et al. (2015b)	EXP1	20	37	Yes; vocabulary	Match/mismatch	Picture-word priming paradigm	Existing and pseudowords	Yes	D	A	600	800	B^*^	LL/M/RL^*^
		EXP2	24	33	Yes; vocabulary	Match/mismatch	Picture-word priming paradigm	Existing and pseudowords	Yes	D	A	200	800	F/C/P^*^	LL/M/RL^*^
4	Cantiani et al. (2017)		20	20	Yes; risk of language impairment	Match/mismatch	Picture-word priming paradigm	Existing and pseudowords	No	P	A	400	1,000	B^**^	RL^**^
5	Cosper et al. (2020)		10	32	No	Match/mismatch	Sound-word priming paradigm	Pseudowords	Yes	D	M	300	400	B	M/LL
6	Duta et al. (2012)		14	18	No	Match/mismatch	Picture-word priming paradigm	Existing and pseudowords	No	D	M	400	600	B	LL/M/RL
7	Forgács et al. (2019)	EXP1	14	18	No	Match/mismatch	Live object naming paradigm	Existing and pseudowords	No	P	A	400	600	P	LL/M/RL
		EXP2	14	18	No	Match/mismatch	Live object naming paradigm	Existing words	No	P	A	400	600	P	LL/M/RL
8	Forgács et al. (2020)	EXP1	18	14	No	Match/mismatch	Live object naming paradigm	Existing words	No	P	A	400	600	P	LL/M/RL
		EXP3	18	14	No	Match/mismatch	Live object naming paradigm	Existing words	No	P	A	400	600	P	LL/M/RL
9	Friedrich and Friederici (2005a)		14	30	No	Match/mismatch	Picture-word priming paradigm	Existing words	No	D	M	400	1,000	P	M
10	Friedrich and Friederici (2005b)		19	47	Yes, risk of language impairment at 30 months^∧^; concurrent vocabulary∧	Match/mismatch	Picture-word priming paradigm	Existing, pseudo- and non-words	No	P & D	M	400	1,200	C	M
11	Friedrich and Friederici (2005b)	EXP1	19	37	No	Match/mismatch	Auditory sentences	Existing words	No	D	M	400	1,200	C/P	LL/M/RL^**^
		EXP2	24	49	No	Match/mismatch	Auditory sentences	Existing words	No	D	M	300	1,200	F/C/P	LL/M/RL
12	Friedrich and Friederici (2008)		14	31	No	Match/mismatch	Picture-word priming paradigm	Pseudowords	Yes	V	M	200	1,000	P	M/RL
13	Friedrich and Friederici (2011)		6	44	No	Consistent/ inconsistent pairings	Picture-word priming paradigm	Pseudowords	Yes	P & V	M	600	900	P	M
14	Friedrich et al. (2015)		12	90	Yes; with/ without nap	Consistent/ inconsistent pairings	Picture-word priming paradigm	Pseudowords	Yes	P & V	M	300	700	F/C/P^**^	M^**^
15	Friedrich et al. (2017)		7	107	Yes; with/ without nap	Match/mismatch	Picture-word priming paradigm	Pseudowords	Yes	D	M	376	710	C/P^**^	M/RL^**^
16	Friedrich et al. (2019)		15	30	Yes; with/ without nap & vocabulary	Consistent/ inconsistent pairings	Picture-word priming paradigm	Existing and pseudowords	Yes	P	M	600	1,000	P^**^	M^**^
17	Friedrich et al. (2020)		15	60	Yes; with/ without nap	Match/mismatch	Picture-word priming paradigm	Existing words	Yes	P & V	M	400	800	P^**^	LL/M/RL^**^
18	Helo et al. (2017)		24	31	Yes; vocabulary	Match/mismatch	Video-word priming paradigm	Existing words	No	D	A	400	700	F^**^	LL^**^
19	Hendrickson et al. (2019)		20	16	No	Match/mismatch	Picture-word priming paradigm	Existing words	No	P & V	M	200	1,000	F, CP, P	LL/M/RL
20	Hirotani et al. (2009)		20	23	No	Match/mismatch	Picture-word priming paradigm	Existing and pseudowords	Yes	D & V	M	800	1,200	F/C	LL
21	Junge et al. (2012a)		9	20	Yes; vocabulary	Match/mismatch	Picture-word priming paradigm	Existing words	Yes	V	M	400	600	B	RL
22	Mani et al. (2012)		14	16	No	Match/mismatch	Picture-word priming paradigm	Existing and pseudowords	No	P & D	M	400	600	F/C	LL
23	Parise and Csibra (2012)		9	28	Yes; with/ without parent uttering the words	Match/mismatch	Word-object priming paradigm	Existing words	No	P & V	A	500	650	C/P^**^	LL/RL^**^
24	Rämä et al. (2013)	EXP2	24	23	Yes; vocabulary	Match/mismatch	Word-word priming paradigm	Existing words	No	D	A	0	600	P^**^	RL^**^
25	Sheehan et al. (2007)		18	17	No	Match/mismatch	video with spoken word /gesture-picture priming paradigm	Existing words	No	D	M	200	600	B^**^	LL/RL^**^
26	Sirri and Rämä (2015)		18	20	Yes; vocabulary	Match/mismatch	Word-word priming paradigm	Existing words	No	P	M	300	500	C/P*	LL/RL*
27	Torkildsen et al. (2006)		20	27	Yes; vocabulary, and ± risk of dyslexia∧	Match/mismatch	Picture-word priming paradigm	Existing words	No	D	M	Multiple time windows	B*	LL*
28	Torkildsen et al. (2007a)		24	17	Yes; ± risk of dyslexia∧	Match/mismatch	Word-word priming paradigm	Existing words	No	D	M	600	800	F/C*	LL*
29	Torkildsen et al. (2008)	EXP1	20	44	Yes; vocabulary	Match/mismatch	Picture-word priming paradigm	Pseudowords	Yes	D	M	200	400	B*	LL/M/RL*
		EXP2^°^	20	44	Yes; vocabulary	Match/mismatch	Picture-word priming paradigm	Existing words	Yes	D	M	400	800	B*	LL/M/RL*
30	Travis et al. (2011)		15	12	No	Match/mismatch	Picture-word priming paradigm	Existing words	No	P & V	N/A	350	550	C	LL

* *Taken from omnibus results;*

** *Taken from results of a single condition;*

° *Authors split analysis into two experiments because of the manner in which the analysis is split in the paper;*

∧ *Results published in an additional paper (see Table 2).*

*N400 selection {P, previous literature; D, data-driven with statistical tests; V, Visual inspection}; Reference electrode {M, mastoids, A, common average}; TW, time window; Distribution Ant/Post, Anterior-Posterior axis {B, broad; C, central, F, frontal, P, parietal} l distribution laterality-axis {LL, left-lateralized; RL, right-lateralized; ML, midline; B, broad}*.

**Table 2:** List of subgroups and condition analyses within papers listed in Table 1.

**Record**	**References**		**Age (months)**	**Analysis type**	**Experimental condition or subgroup specification**	**N400 TW onset**	**N400 TW offset**	**Distribution: ant-post**	**Distribution: laterality**
2	Borgström et al. (2015a)	EXP1	20	Between-and-within-subjects	Regular pictures	500	900	P	LL/M/RL
					Silhouette pictures high vocab^*^	500	900	C	M
					Silhouette pictures low vocab^*^	Null effect	Null effect	Null effect	Null effect
					Detail picture	Null effect	Null effect	Null effect	Null effect
		EXP2	24	Between-and-within-subjects	Regular pictures	500	900	P	LL/M/RL
					Silhouette pictures high vocab^*^	500	900	C	LL/M/RL
					Silhouette pictures low vocab^*^	Null effect	Null effect	Null effect	Null effect
					Detail picture	Null effect	Null effect	Null effect	Null effect
3	Borgström et al. (2015b)	EXP1	20	Within-subject	Pseudowords	Null effect	Null effect	Null effect	Null effect
					Real words	400	800	P	LL/M/RL
		EXP2	24	Within-subject	Pseudowords	400	1,000	C,P	RL^*^
					Real words	400	800	P	LL/M/RL
4	Cantiani et al. (2017)		20	Between-and-within-subjects	Real words FH (not at risk)	400	1,000	C	RL
					Real words FH+ (at-risk)	400	1,000	B	LL/M/RL
					Pseudowords FH (not at risk)	100	700	P^*^	LL/M/RL
					Pseudowords FH+ (at-risk)	400	700	B^*^	LL/M/RL
10	Friedrich and Friederici (2004)		19	Between-subjects	Real words-high comprehenders	300–400; 600	1,000	B	M/RL
					Real words-low-average comprehenders	1,200	1,300	B	L
	Friedrich and Friederici (2006)		19	Between-and-within-subjects	Real words-low risk L.I.	250	1,200	B	B
					Real words-high risk L.I.	Null effect	Null effect	Null effect	Null effect
					Pseudowords vs. non-words-low risk L.I	250	1,100	B	B
					Pseudowords vs. non-words-high risk L.I	Null effect	Null effect	Null effect	Null effect
	Friedrich and Friederici (2010)		12	Between-subjects	Real words-high producers	500	1,000	C,P	RL
					Real words-low-average producers	Null effect	Null effect	Null effect	Null effect
14	Friedrich et al. (2015)		12	Between-subjects	Object meaning no nap	Null effect	Null effect	Null effect	Null effect
					Object meaning nap	200	500	B	LL/M/RL
					Category meaning no nap	Null effect	Null effect	Null effect	Null effect
					Category meaning nap	300	700	F/C/P	M
15	Friedrich et al. (2017)		7	Between-subjects	No nap	Null effect	Null effect	Null effect	Null effect
					Short nap	Null effect	Null effect	Null effect	Null effect
					Long nap	376	710	C/P	M/RL
16	Friedrich et al. (2019)		15	Between-subjects	No nap	Null effect	Null effect	Null effect	Null effect
					No spindle density increase; nap	Null effect	Null effect	Null effect	Null effect
					Spindle density increase; nap	600	1,000	P	M
17	Friedrich et al. (2020)		15	Between-subjects	New objects (nap and no nap)	400	800	P	LL/M/RL
					Old objects; no nap	400	800	P	LL/M/RL
					Old objects; nap	Null effect	Null effect	Null effect	Null effect
18	Helo et al. (2017)		24	Between-subjects	High producer	400	700	F	LL
					Normal-to-low producer	550	700	F	RL
22	Mani et al. (2012)		14	Within-subjects	Mispronunciations	400	600	F/C	LL
					Non-words	400	600	F/C	LL
23	Parise and Csibra (2012)		9	Between-subjects	Mother-speech	500	650	C/P	LL/RL
					EXperimenter-speech	Null effect	Null effect	Null effect	Null effect
24	Rämä et al. (2013)	EXP1	18	Between-subjects	High producer	Null effect	Null effect	Null effect	Null effect
					Low producer	Null effect	Null effect	Null effect	Null effect
		EXP2	24	Between-subjects	High producer	200	400	P	RL
					Low producer	Null effect	Null effect	Null effect	Null effect
25	Sheehan et al. (2007)		18	Within-subject	Picture	200	600	B	LL/RL
					Gesture	200	600	F/C	LL/RL
26	Sirri and Rämä (2015)		18	Between-subjects	High producer	300	500	C/P	B
					Low producer	300	500	C/P	B
27	Torkildsen et al. (2006)		20	Between-subjects	High producer (within and between category violations)	600	700	B	LL/RL
					Low producer (within and between category violations)	1,100	1,250	B	LL
	^∧^Torkildsen et al. (2007b)				Additional group of infants at risk of dyslexia^∧^	Null effect	Null effect	Null effect	Null effect
28	Torkildsen et al. (2007b)		24	Between-subjects	Typically developing	400	700	F	M
					Additional group of infants at risk of dyslexia^∧^	600	700	B	LH
29	Torkildsen et al. (2008)		24	Between-and-within-subjects	High producer-familiar words	400	800	B	B
					Low producer-familiar words	400	800	C/P	M
					High producer-novel words	200	800	CP	Midline
					Low producer-novel words	400	800	C/P	M/RL

*Studies are sorted by the original record that published findings (see Table 1); the second column with Author and publication lists the source in which the subgroups or within-subjects are analyzed.*

* *Results from a single TW interaction;*

∧ *Results published in an additional paper.*

*TW, time window; Distribution Ant/Post, Anterior-Posterior axis {B, broad; C, central, F, frontal, P, parietal}; distribution laterality-axis {LL, left-lateralized; RL, right-lateralized; ML, midline; B, broad}; FH, familial risk of language impairments; L.I., language impairments Table 1*.

All studies tested semantic processing using a priming paradigm, although they differed in what was the prime and what was the target. In most samples (*n* = 28/35) the N400 effect was elicited when infants saw a visual stimulus as the prime (picture: *n* = 23; real object: *n* = 4; video: *n* = 1), and heard an acoustically presented word as the target. Usually the visual prime was still visible while the target was presented. A couple of studies reversed this, having an auditory word serve as a prime, and a visual stimulus as the target. Due to the fleeting nature of speech, this entailed that the prime was no longer present at target onset, possibly affecting the latency of the N400 (see also Sheehan and Mills, 2008). Other studies (*n* = 5) solely relied on auditory tokens, such as a sentence context or single word context, or testing the association between environmental sounds and words.

The linguistic N400 effect was predominantly elicited in contexts in which the same target either matched or did not match the prime (match/mismatch paradigms that test semantic incongruity, *n* = 33/35 samples), although it was also observed in novel-word training studies in which words were either constantly or randomly paired against novel objects. Table 1 further lists the kind of words presented: known words, pseudowords, or non-words. There were 19 samples that were tested solely with typically existing familiar words, whereas there were 6 samples that focused solely on novel word-learning, and 10 samples that listened to both existing and pseudowords. Of those studies testing novel word learning, most used a training-and-test phase within the same session, although there were some that manipulated delays of the test phase.

For the 34 samples for which EEG was measured, the N400 components were predominantly elicited when the signal was re-referenced to the mastoids (*n* = 22/34), although average reference has also been used (*n* = 12/34). In all studies, the N400 for each experimental condition was quantified as the mean amplitude in a given time window or series of adjacent time windows; there were no studies reporting peak latency (the moment in time when a component reaches its maximum). In half of the analyses the N400 was recognized and analyzed using a data-driven approach (e.g., guided by visual inspection or using statistical analyses in which the time-window was not a priori defined), whereas in the other 18 samples researchers based their statistical analysis on previous literature or a combination of previous literature and visual inspection. An independent *t*-test analysis revealed that analyses that used a data-driven approach to identify time windows usually appeared in older publications (mean year of publication is 2011) than studies that rely on pre-existing literature (mean year of publication is 2015; *T*_(34)_ = 3.28, *p* = 0.002). Thus, perhaps not surprisingly, studies mainly used an explorative approach when semantic priming paradigms were still novel to test linguistic processing in infants.

Finally, we examined the number of samples in which correspondences were observed between the N400 and other individual characteristics. Although in 17 samples there was no examination of within-group variation, in the remaining samples there was: 13 studies noted correspondences with vocabulary size or (increased risk of) language impairments including dyslexia; three that linked it solely to amount of sleep in between training and test; one to a combination of sleep and vocabulary; and one to differences in testing environments. Thus, there was ample ground to inspect whether latency and distribution of the N400 are sensitive to individual variation in vocabulary size.

In what follows next, we considered how age, experimental designs, and individual differences impacted the characteristics of the N400. Given the imbalance in studies using a visual stimulus as prime vs. studies that employed an auditory stimulus as prime, we could not systematically investigate whether temporal structure impacted the N400. Arguably, integration or lexical access is more difficult when the prime is no longer present once the target is presented, which could impact the timing or distribution of the N400 (Sheehan and Mills, 2008). This is why we decided to focus on the majority of priming studies using a similar prime-target set-up: the 28 samples that employed a visual stimulus as prime, and auditory word as target.

### Timing and Distribution of the N400 Modulated by Age?

When we examined the latency of the N400 effect in the first 24 months of life, we saw there was quite some variation, particularly in the offset. In some studies, the N400 priming effect time window appeared adult-like [i.e., 200–600 ms (Kutas and Federmeier, 2011); in infants e.g., Sheehan et al., 2007; Forgács et al., 2020], but in the majority of studies both the onset and offset of the reported infant-N400 effects occurred later. For one data-driven analysis that used 100 ms intervals from 200 to 1,200 ms we could not point to one single stretch of significant adjacent time windows but identified instead multiple shorter but not adjacent intervals (Torkildsen et al., 2006); therefore, this one record is omitted here. We therefore carried out our statistical descriptives based on 27 analyses.

**Figure 2:**
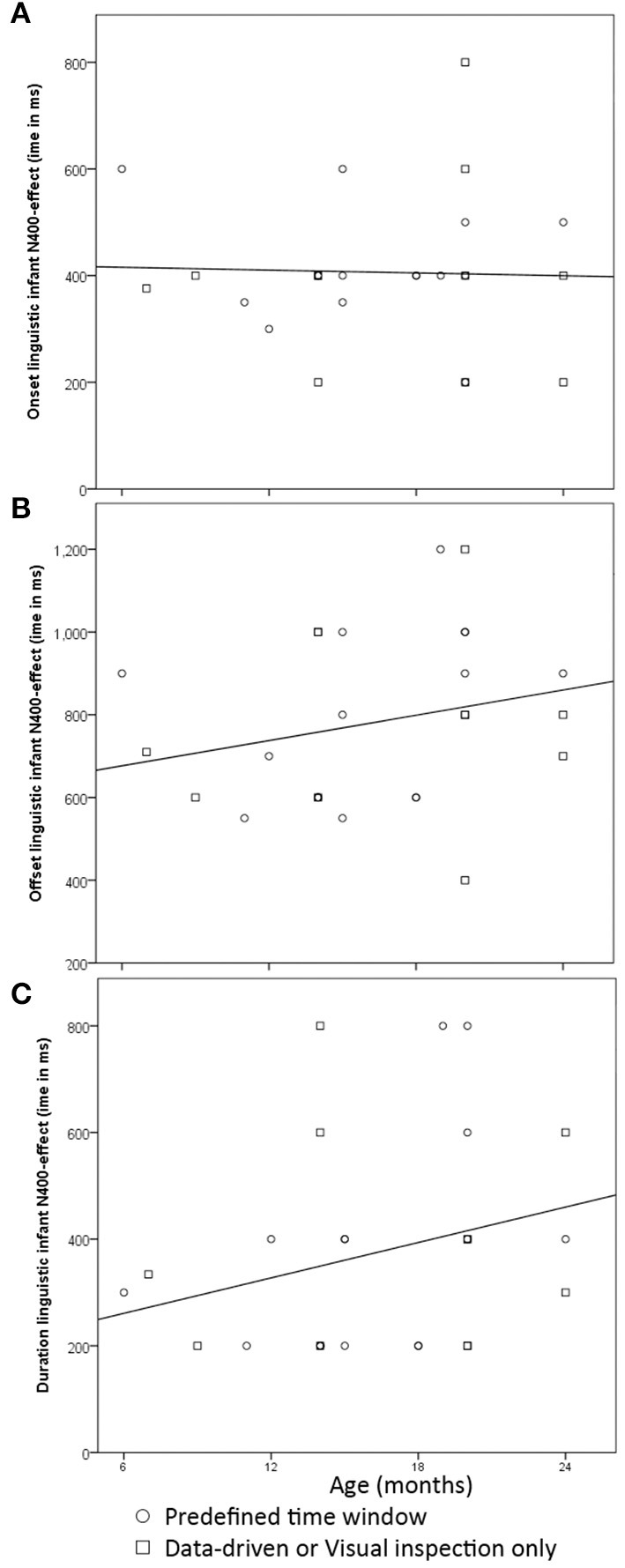
Scatterplots with the time window for the N400 as a function of infant participants' ages in months: **(A)**—onset; **(B)**—offset; **(C)**—duration of the time window all in ms.

Figure 2 provides scatterplots of these 27 analyses and gives an indication of how the onset, offset and duration of the N400 priming effect are modulated by age. We distinguished between those analyses based on pre-defined time windows and those that focused on a data-driven approach, as data-driven analyses might be heuristically different in how they approach time window selection than those analyses that rely on pre-defined choices. There were 11 analyses that used a data-driven or visual inspection, and 16 analyses that relied on a pre-defined choice of time windows. We reported correlations between age and each of the time window characteristics for the total of 27 analyses.

When we examined the onset of the N400, most analyses (*n* = 16/27) included amplitudes obtained in a time window starting at 400 ms, or within 50 ms prior. The choice of time onset did not change with age [*r*_(27)_ = −0.032; *p* = 0.88]. There was more variation in the offset of the N400, and correspondingly, the total length of the time window. Its offset was often till 600 (*n* = 7) or 800 ms (*n* = 4), but several analyses also included time windows up to 1,000 (*n* = 5) or even 1,200 ms (*n* = 2) after target onset. There were insignificant trends with age: with age the offset of the time window increased slightly [*r*_(27)_ = 0.24; *p* = 0.23], thereby increasing total length of the time window [*r*_(27)_ = 0.27; *p* = 0.18]. These patterns did not change significance (*p* < 0.05) when we examined each of the statistical approaches separately. What was striking was the wide range of variation in the time windows in which the N400 priming effect occurred in infants, which is more notable from 12 months onwards. That is, reported offsets ranged from 550 to 1,000 ms after word onset in infants between 12 and 18 months old, and varied from 400 to 1,200 ms after word onset between 19 and 24 months. The amount of time window variation reported between 19 and 24 months was in contrast with individual studies finding identical N400 effect time windows for infants of different age groups within the second half of the second year (Friedrich and Friederici, 2005b; Borgström et al., 2015a). That is, 20- and 24-month-olds in Borgström et al. (2015a) both showed an N400 priming effect ranging from 400 to 900 ms after word onset (but see Borgström et al., 2015b). Similarly, in Friedrich and Friederici (2005b) the reported N400 effect had a time window of 400–1,200 ms in both 19- and 24-month-olds. Whereas these two studies individually suggested that infants between 19 and 24 months old initiated semantic integration mechanisms around the same time and required the same amount of effort to integrate a verbal prime with a given context, the overall results of all studies combined further underscored that age alone cannot explain this variation in latency.

**Figure 3:**
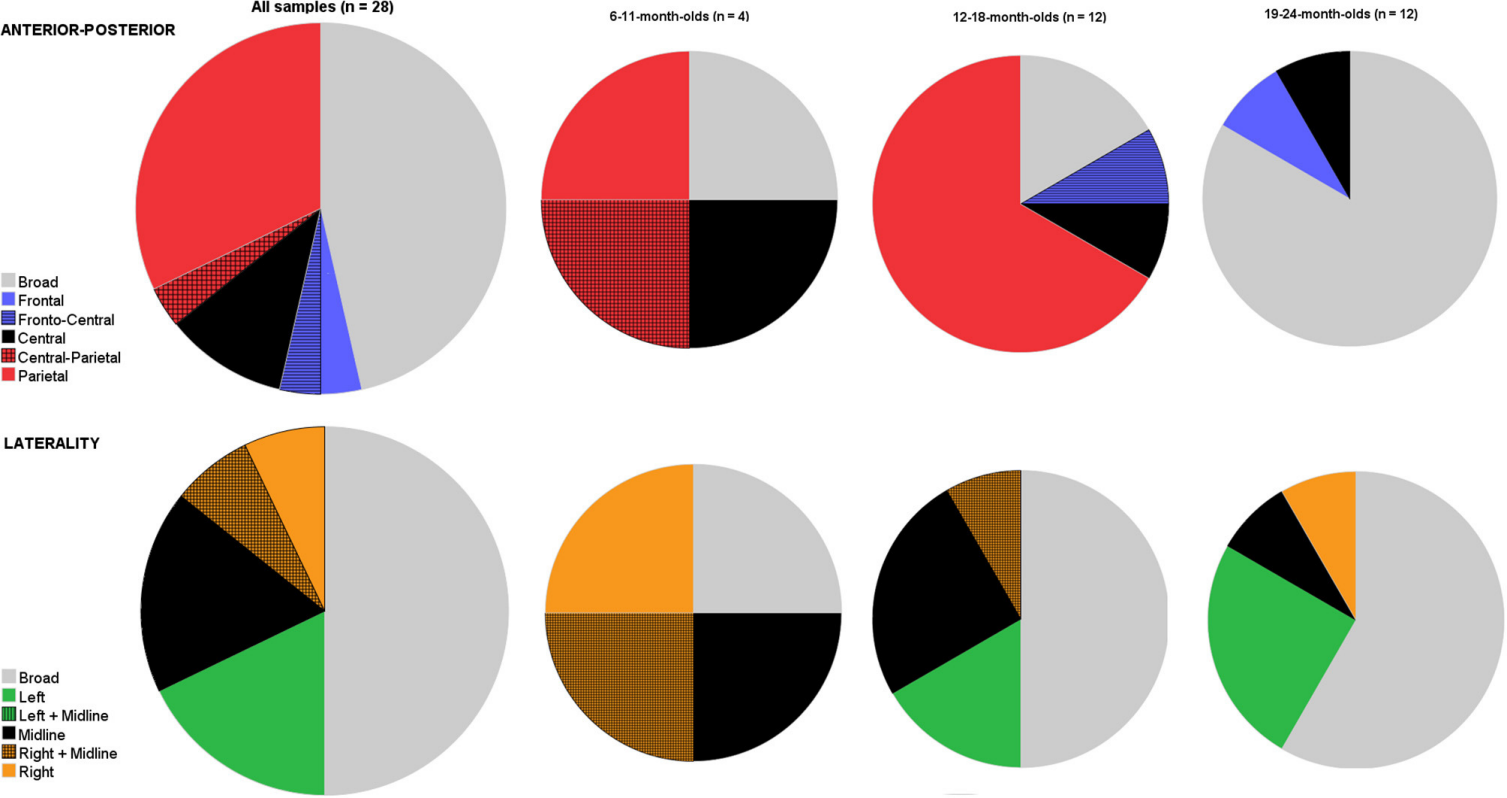
Pie charts depicting the variation in studies on where on the scalp it is that the N400 priming effect is present (top row: anterior-posterior axis; bottom row: lateral axis) shown for all 28 visual object-word priming studies (age: 6–24-month-olds), and subdivided into three smaller age bins (6–11-month-olds; 12–18-month-olds; 19–24-month-olds). For both axes, there is also a broad category possible, indicating that there is no statistical evidence that the N400 is differently distributed across this axis.

Next, we examined whether age contributed to the distribution of the N400. Remember that we considered effects only to have a focal distribution when omnibus ANOVAs warranted an interaction between condition and distributional factor(s). Figure 3 shows pie charts for the distribution factors, both for the anterior-posterior axis (top) and the laterality-axis (bottom). We first plotted findings for all ages, and to allow for examining variation by age, then split these into three age bins: 6–11-month-olds (*n* = 4); 12–18-month-olds (*n* = 12), and 19–24-month-olds (*n* = 12). As with the latency, there was heterogeneity in where the N400 effect was observed. For both axes, topographical distribution of the infant-N400 effect varied from local and adult-like (i.e., centro-parietal, Kutas and Federmeier, 2011) to more widely spread around the scalp.

Concerning the anterior-posterior axis, most reported locations of the effect was a broad distribution (*n* = 13/28) or at central and/or parietal (*n* =13/28). Finally, there were also some studies that reported the effect to be present on only frontal (*n* = 1/28) or frontal plus central electrodes (*n* = 1/28).

When comparing development in distribution across ages, we observed that there was variation in all age bins. Arguably, the youngest age bin was rather small. Nevertheless, when we compared focal vs. broad distributions for all three age bins using this simple categorical contrast, a chi-square test indicated that the age groups differed in the proportion of broad distributional effects [χχ${}_{\left(2,\;\text{n}=28\right)}^{2}$ = 11.6, *p* = 0.003]: the N400 appeared predominantly as a broad distribution in the 19–24-month-olds (*n* = 10/12, 83.3%), while as a focal distribution in the younger age groups (that is, a broad distribution was only present in 1/4 and 2/12 analyses of 6–11- and 12–18-month-olds, respectively). Figure 3 further clearly shows the involvement of broader areas including frontal areas in semantic processing in the oldest age bin. That is, whereas 3 out of 12 (25%) observed N400 components in analyses from 12 to 18-month-olds included broad or frontal sites, frontal areas were involved in 9 out of 11 (81.8%) reported N400 effects in analyses from infants aged between 19 and 24 months. Conversely, the N400 component appeared more often focally distributed to centro-parietal regions in 12–18-month-olds (*n* = 9/12, 75%) than was the case with analyses from older infants (2 out of 12, 18.2%). Thus, it appeared that N400 priming effects included frontal sites more often in the oldest age group than in the younger age groups.

Finally, we inspected hemispheric differences (Figure 3 bottom row). Again there was some variation, but this was similar across ages. Half of the analyses (*n* = 14/28) reported finding the N400 effect to be more pronounced in the left or right hemisphere or localized to midline electrodes; whereas others reported it to be broadly distributed. However, from those studies reporting a more focal distribution, no consistent picture emerged: Five studies reported it to be more localized to the left hemisphere; five for it to be present only on midline electrodes, and another four to be more pronounced to the right hemisphere. Thus, whether there was a hemispheric preference for the N400 effect in infants was far from consistent. Also, age did not appear not very informative: results were mixed in all age bins. When we carried out a similar Chi-square test with broad vs. focal distributions, the proportion of distributions did not change as a function of age groups [χ${}_{\left(2,\;\text{n}=28\right)}^{2}$ = 1.33, *p* = 0.51].

### Timing and Distribution of the N400 Modulated by Word-Type?

Even within the visual stimulus-spoken word priming paradigm, studies further differed besides age in their experimental designs, most notably in their choice of spoken word stimuli. That is, some studies tested existing semantic knowledge, while others relied on novel word learning. We reasoned that testing knowledge of novel words (e.g., word-object pairings usually acquired within the same recording session) would be more challenging than testing knowledge of typically early and familiar words, which rely on ample pre-exposure on various object-word pairings. As a simple index of task difficulty we therefore compared analyses based on novel word processing (*n* = 15) with those that were solely obtained in existing words -paradigms (*n* = 13) on characteristics of the N400 component. These analyses did not differ in the ages tested [*t*_(26)_ = −1.35, *p* = 0.19].

With respect to latency, again there was variation, which was similar for both sets of analyses with respect to offset and duration of the N400, but there appeared more variation in the onset of the N400 in analyses on novel words (Levene's test: *F* = 5.52, *p* = 0.027). Simple *t*-tests revealed that differences between sets of analyses were neither significant for the onset [*t*_(25)_ = 0.39, *p* = 0.70], for the offset [*t*_(25)_ = 0.61, *p* = 0.55], nor for the duration [*t*_(25)_ = 0.39, *p* = 0.70].

**Figure 4:**
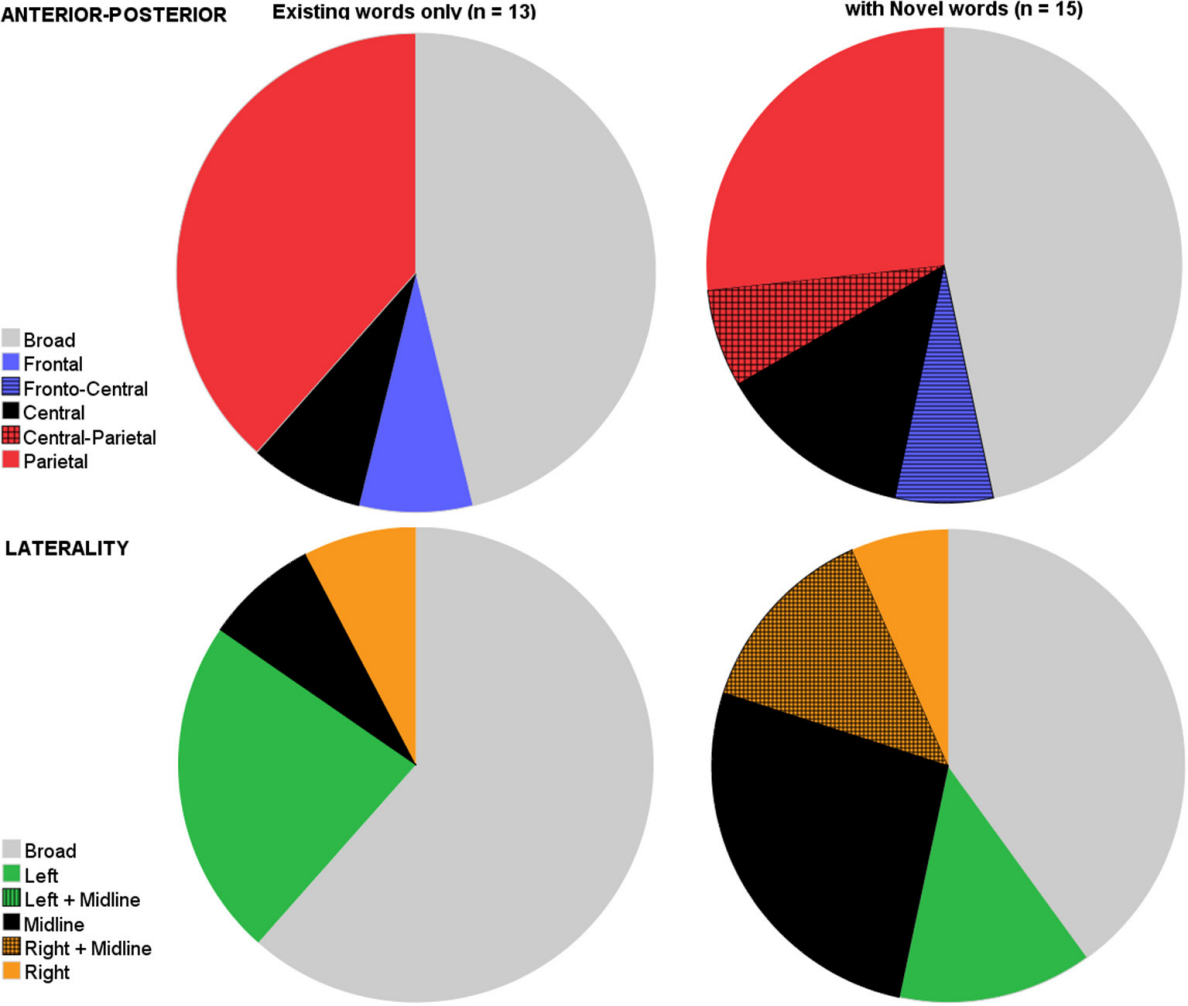
Pie charts depicting the variation in studies on where on the scalp it is that the N400 priming effect is present (top row: anterior-posterior axis; bottom row: lateral axis) split by studies that either solely tested semantic processing of existing early words (left), or (also) included novel words (right).

We then compared the two sets of analyses based on the distribution factors. As Figure 4 shows, both sets showed similar broad distributions, both for the anterior-posterior axis, as well as for the laterality-axis. With respect to the anterior-posterior axis, components elicited with novel words showed a comparable proportion of broad distributions (46.7%) as components elicited with only existing words [46.2%, *t*_(26)_ = 0.03, *p* = 0.98]. Similarly, for the laterality axis, analyses based on existing words (61.5%) did not differ from analyses on novel words in their proportions of broad distributions spanning both hemispheres [40%, *t*_(26)_ = −1.12, *p* = 0.27].

### Timing and Distribution of the N400 Modulated by Individual Differences?

Studies used different designs to inspect the relationship between the ERP component N400 at the individual level: correlations, between-groups, and within-groups designs. Each type of design is informative about whether the N400 changes as a function of individual characteristics.

First, when we turned to those studies that reported correlations between one index of the N400 and other variables such as vocabulary measures, we cannot compare how latency and distribution factors varied in individuals: The N400 has been quantified the same way for all individuals using a fixed definition of time window and distribution. All that varied was reflected in changes in the amplitude. Nevertheless, the sign of the correlation informed us whether the linguistic N400 effect increased or decreased with other characteristics of the individual. In two studies the correlation was assessed against a child's concurrent vocabulary size (Junge et al., 2012a; Borgström et al., 2015b). Here, we observed a pattern that the N400 effect usually increased with vocabulary size.

Studies that used a within- or between-subjects design prove more informative about how the latency and distribution of the N400 is modulated across different participants (i.e., creating subgroups resulting in a between-participants design) or across different situations (within-subjects design). Table 2 lists the samples that examined variation in the elicitation of the N400 using a between-subjects design (*n* = 14), a within-subjects design (*n* = 4), or a combination of the two (*n* = 5).

We first examined the studies using a between-subjects condition, that is, those comparing subgroups of infants of the same age who differ in some additional respect with the same test phase. Of those studies using a mixed design, these usually compared the effect of vocabulary size across infants on multiple situations that could elicit the N400. For instance, Borgström et al. (2015a) used a median split to compare 20-month-olds with a high or low expressive vocabulary on three versions of the semantic congruity paradigm: with the picture being either a complete representation of an object, or depicting only the silhouette, or just a few details from the object. The majority of studies using a between-subject design focused on language proficiency (*n* = 14), usually relying on a quantitative measure of vocabulary size (*n* = 9). Other studies examined language proficiency by creating subgroups based on whether infants were at elevated risk of language impairments (*n* = 2) or dyslexia (*n* = 2). For instance, Friedrich and Friederici (2006) grouped 19-month-olds retrospectively based on whether or not these infants were considered to be at risk for a language impairment according to expressive language scores collected at 30 months.

We reasoned that language proficiency would be another way to examine the impact of experimental designs difficulty. Presumably infants who are more advanced than their peers in language development would be more sensitive to semantic priming, and hence show a more mature N400. When we zoomed in on those studies including measures on language proficiency, we observe that the N400 is often but not always modulated by language differences: in six subgroup comparisons language-groups appeared similar. Nevertheless, the majority of cases reported differences (*n* = 15/21; condition we listed each within-subject condition separately). Out of these 15 cases, there were seven comparisons that reported an absence (null-effect) for one group, but an N400 effect for the other group; of these comparisons it was always the more proficient group in which the N400 was present. Finally, there were eight comparisons, in which the N400 was elicited in both groups, but somewhat differently per language-group. With respect to latency differences, in six comparisons the effect started earlier for the group more proficient in language skills. For instance, the onset of the N400 was 400 ms in a high-producer group, but was delayed up to 550 ms in a low-average-producer group (Helo et al., 2017). Further, all eight comparisons pointed to differences in distribution, but no clear picture emerged to differentiate higher from lower proficient samples. All kinds of patterns appeared possible with respect to hemispheric differences. For instance, while one comparison showed that in a higher-proficient sample the effect was distributed in the left hemisphere whereas it was in the right hemisphere for a lower-proficient sample (Helo et al., 2017), other studies suggested that the N400 in lower-proficient samples was present on left electrodes (Friedrich and Friederici, 2004; Torkildsen et al., 2007b). There was less variation in differences in the anterior-posterior axis, but still results were inconsistent: three comparisons listed a broader distribution for the lower-proficient than for the higher-proficient samples, and another four reported no differences.

Furthermore, Friedrich and colleagues used a between-subject design to identify another factor of interest that modulated the presence of the N400: sleep. In a series of studies, they compared infants that either had no or a short nap prior to testing with infants who had a longer nap between learning novel words and testing. They showed that only infants with a longer nap, and with deeper sleep characteristics, were the ones in which the N400 could be elicited (cf. Friedrich et al., 2020).

Next, we examined the studies that reported on various situations in which the N400 is elicited (1 between-subject condition; 4 within-subject condition; 4 both within-and between sample condition; total *n* = 9). As within-subject studies test the *same* infants in different situations, they offer the advantage that any variation in N400 characteristics could not be explained by factors inspected above, that is, age or in language proficiency. We reasoned that any kind of familiarity (be it with pictures, with the speaker, or with words) would facilitate semantic processing, and therefore increase the likelihood that an N400 would be obtained. Indeed, familiarity with complete pictures rather than parts of pictures resulted in shorter onsets (Borgström et al., 2015a). For spoken words vs. iconic gestures we noticed no differences in latency, but a more frontal-central distribution for gestures (Sheehan et al., 2007). Familiarity with the speaker explained the presence vs. absence of the N400 (Parise and Csibra, 2012). Yet when we considered whether familiarity with the word explained variation in N400 characteristics (e.g., comparing existing words with pseudowords), results were mixed, both with respect to its latency and to its distribution. There were some studies that reported null-effects for pseudowords (Borgström et al., 2015b with 20-month-olds), or delayed N400 effects for pseudowords (Borgström et al., 2015b with 24-month-olds), whereas others reported a delayed N400 for existing words relative to pseudowords (Torkildsen et al., 2008; Cantiani et al., 2017; for high producer group only). Similarly, distribution effects appeared to be more focally distributed to the right hemisphere for pseudowords in one sample (Borgström et al., 2015b), whereas it can be more right-lateralized for existing words in another sample (e.g., Cantiani et al., 2017, for infants with low risk of language impairments).

Finally, there were two records that compared the amount of violation in incongruous conditions using a within-subject semantic-priming paradigm. Torkildsen et al. (2006) tested whether N400 responses differed depending on whether the violation concerned a within-category violation or a between-category violation: results showed that the incongruity response was earlier and larger for between-category violations than for within-category violations. Mani et al. (2012) used in their incongruous conditions pseudowords that were either minimally mispronounced by changing a vowel from the correct representation, or being completely different: results showed that both conditions elicit similar left-frontal N400 components.

## Discussion

The aim of this review was to characterize developmental changes in the N400 indexing lexico-semantic processing in the first 2 years of life, and to provide guidelines for quantifying the latency and distribution of the N400 effect in infant research. Because the latency, amplitude, and distribution of ERP components are sensitive to even subtle manipulations in the stimuli and experimental designs, we chose to limit our statistical analyses to the 28 published analyses on the linguistic N400 elicited in visual stimulus-word match-mismatch paradigms. This N400 priming effect has been observed as early as 6 months of age with a minimal pairing of objects and novel words (Friedrich and Friederici, 2011). In school-aged children, with a different paradigm (listening to semantically congruent or incongruent sentence endings), the peak latency of the N400 decreased from 619 ms at 5 years of age until 498 ms at 13 years of age after which it was stable, and the distribution became more focal over posterior regions with increasing age (Holcomb et al., 1992). We also based this hypothesis on similar developmental tracks of ERP components, particularly related to latency, such as the infant–like N290 component (Halit et al., 2003) that is comparable but delayed compared to the adult-like N170 (Bentin et al., 1996; Eimer, 2011). Thus, we predicted a similar trend might be observed in the first 2 years. Below we discuss our findings of the infant N400 in terms of latency and distribution, separately.

### The Latency of the Infant N400

Our analyses of the infant data provided weak evidence that the onset of the match-mismatch N400 decreases and the offset increases with increasing age, but these correlations failed to reach statistical significance. Although N400 peak latencies are relatively stable in young healthy monolingual adults (Kutas and Federmeier, 2011), they decrease with increasing age in children (Holcomb et al., 1992). The peak latency and onset latencies of the N400 have been taken as indicators of processing speed (Joyal et al., 2020). In infants, intermodal (visual picture—spoken word) eye-tracking studies find that processing speed decreases with increasing age as well as with vocabulary size in the first 2 years of life (Fernald et al., 1998, 2006). Therefore, it seems reasonable to assume that N400 peak latency and onset latency of the priming effect should show similar trends in ERP picture—word priming studies in the infant studies reviewed here. Ideally we had analyzed variation in peak latencies; however, none of the infant studies reported peak latencies, presumably because it is difficult to pinpoint latencies for broad infant components that are long-lasting, such as is the case with the infant-like N400 (DeBoer et al., 2007). Instead we turned to how researchers defined their time windows. One possible explanation for the lack of statistically significant correlations with decreasing N400 latency and increasing age lies in variability across the methods for quantification. In the present study, the approach taken was to use the time windows specified in each analysis for statistically reliable differences in N400 amplitudes. The onset of the N400 effect was assumed from the choice of time windows researchers had considered. We adopted this procedure because systematic analyses of the onset and offset of the effect using sequential overlapping short epochs were not consistent across studies. However, most studies did not systematically test its onset and/or offset (nor were they designed to do so). Therefore, albeit the lack of consistency in the literature as it pertains to the methodology of selecting the onset/offset of the N400, we are bound to only describing the literature and comparing the onset/offset times in a general manner. Thus, our results on this should be considered accordingly.

Another consideration is that the early (200–400 ms) and later (400–600 ms) time windows might represent functionally distinct processes with different developmental trajectories. In adults the picture-word paradigm has been used to examine a phonological mismatch (PMN) starting at 200 ms, followed by the N400 associated with semantic integration in adults (e.g., Desroches et al., 2009; Newman and Connolly, 2009). In infants, studies using picture—word match/mismatch paradigms showed early ERP sensitivity to mispronunciations of the initial consonant (Duta et al., 2012) or middle vowels from 200 to 300 ms and 400 to 600 ms (Mani et al., 2012). However, unlike adult studies, mispronunciations and non-words did not show separable ERP effects as there was no condition in which there was a semantic but no phonological mismatch and visa-versa. It is possible that younger age groups might be sensitive to phonological mismatch, as demonstrated by Mismatch Negativity (MMN) studies, but do not show a lexical semantic N400 effect until a critical mass in vocabulary is achieved (cf. Junge et al., 2012a).

Moreover, while the onset of the N400 can be difficult to characterize as it might interfere with the PMN, similarly its offset is also hard to define. Some studies consider the latency to have a longer duration than is typically present in adults (e.g., Torkildsen et al., 2006, lasting up to 1200 ms), whereas others consider this to be an additional and distinct slow wave (“late posterior negativity;” Sirri and Rämä, 2015). Thus, there remains a need for studies to systematically report their onsets and offsets.

### The Distribution of the Infant N400

Age-related changes in the distribution of the N400 effect were also observed, with more focally distributed N400 effects in the first 18 months of life, and a more broadly distributed effect in 19–24-month-olds. Although left, right, and bilateral distributions of the N400 priming effect were observed, there were no consistent trends observed with increasing age. Experimental designs and vocabulary size also introduced variability in these effects.

Regarding age related changes in the distribution of the N400, the results proved to be inconclusive as there was a lack of consistency in where and when the N400 effect was measured and reported. A consistent trend from more broadly distributed to more focal or lateralized as occurs in older children was not apparent. It could be that age marks itself as the more additional involvement of frontal electrodes. Additional involvement of frontal electrodes has reported before in developmental populations (Holcomb et al., 1992). Frontal areas are commonly found to be related to the N400 effect in children and can still be found in children up until 13 years of age (e.g., Atchley et al., 2006; Henderson et al., 2011). In previous studies, involvement of frontal areas during the N400 effect in infants has been associated with image-specific processing and with increased attentive demands (Friedrich and Friederici, 2004, 2005a; Torkildsen et al., 2006). In adults, some studies also report the involvement of frontal distributions. As different distributions have been taken to represent differences in underlying neural generators, researchers have hypothesized what could explain these frontal distributions. Some postulate that these more frontal distributions reflect word processing indexing familiarity rather than recollection processing (Rugg and Curran, 2007), whereas others believe they reflect the addition of visual processing resulting in facilitated conceptual processing (Willems et al., 2009). If word familiarity contributes to the infant-like linguistic N400 effect being present on frontal electrodes, then one would expect frontal N400s to be more present in novel word studies than in existing word studies; but we found no such pattern.

However, any observations of distributional changes with age could also stem from different explanations. One issue is that movement artifacts can be problematic in infant ERP data, and might affect some electrodes more than others, which can further interact with age. Infants aged 19–24 months are more likely to sit up by themselves, whereas infants aged 12-months and younger might lean up against the parent or in a car seat, making recordings of more posterior sites less reliable. Also, because of time constraints, infant ERP studies may use fewer electrode sites and the (reference) sites chosen are not always consistent across different labs. There might also be more noise in the data, as impedance levels are usually set higher in infant studies than in adults, and require fewer number of trials to create grand means in the ERPs. Finally, it is important to realize that we draw our observations from analyses in the literature, whereas these original studies were not designed to test distributions of the infant-like N400 in a systematic way. There is great heterogeneity across studies in the number of electrodes recorded during sessions, and not all electrodes are included in the analyses. Thus, for practical reasons, examining focally distributed ERP effects might be observed with some electrode montage configurations and not with others.

### The Amplitude of the Infant N400

The literature with semantic-priming reports the infant N400 to have a negative amplitude for incongruous words relative to congruous words. Correlations of N400 amplitudes with other measures such as vocabulary have been used to examine individual differences. The results suggest that the N400 amplitude increases as a function of vocabulary size when age is held constant. Yet, there are only a few studies that relied on correlations; most studies used a simple contrast to create subgroups (e.g., a between-subjects design) to inspect how latency and distribution of the N400 are modulated by other characteristics from the same individuals. Therefore, due to limited number of studies in the current analysis, further research is needed in order to determine whether increases in amplitude correspond to language proficiency.

### Limitations

One limitation is that we focused our analyses mainly on those analyses obtained in priming paradigms, and because the majority used a visual stimulus-spoken word as target paradigm, we focused on this subtype. Yet, the N400 can also be elicited in studies that do not require visual processing (e.g., Torkildsen et al., 2007a; Cosper et al., 2020). Arguably, due to the fleeting nature of spoken words, it is a different situation as the prime is no longer present to guide the subsequent processing of the target word. However, we do not know whether such changes in temporal structure modulate the timing and distribution of the N400 (but see Sheehan and Mills, 2008) or its amplitude (but for adults, see Cosper, 2020). Even within the same visual stimulus-spoken word paradigm, there is variation in terms of latency and distribution. There is one study that manipulated the timing interval between visual prime and spoken word target, and results revealed only differences in the N200 and late positive waves, but not in the N400 (Sirri and Rämä, 2015).

Another limitation is that we took a component-perspective rather than a paradigm-perspective. That is, we reviewed and contrasted studies that reported positive evidence of the N400 as an index of lexical processing; thereby omitting studies with null-results or with different components. Indeed, whereas some infant paradigms such as the object-word mapping paradigm (in which words are either congruent or incongruent with the objects) typically elicited an N400 similar as in adults, there are other paradigms in which adults typically would show an N400, while infants show a different component. For instance, infants did not show N400 effects in studies without a priming paradigm, in which they only listened to known and unknown words (i.e., pseudo- or non-words) (Mills et al., 1993, 1997, 2004, 2005b; Torkildsen et al., 2009; Parise et al., 2011; Obrig et al., 2017). The N200–500 component reflects familiarity with the word form, without requiring a pairing to a referent (Kooijman et al., 2005; Junge et al., 2012b). It signals that amplitude becomes more negative in the time window 200–500 ms from word onset for familiar words relative to unfamiliar words (Mills et al., 1993, 1997, 2004; Friedrich and Friederici, 2005a). That is, familiar words elicit a larger negativity (rather than a larger positivity) compared to incongruent unfamiliar words, as opposed to the N400, which is more negative for incongruent or unfamiliar relative to congruent words. Thus, despite the suggested commonality in their labelling as both components start with the N for negativity, these components actually result from different control conditions as their point of reference. Had they chosen a similar control condition (say familiar or known words), there would have been a shift in polarity. Hence, the N200–500 overlaps with the N400 in timing, but has a different polarity, and often is present maximally with a left-temporal or fronto-central distribution. Taken together, in the infant literature, the paradigm under study may influence whether an N400 or an N200–500 is elicited, but it is far from clear which component is elicited under which circumstances.

Similarly, the choice of word-form within the experimental paradigms may also influence lexical-semantic processing. In adults, pseudowords show a more negative waveform compared to known words, showing that it is easier to access a real word in the mental lexicon compared to a pseudoword which does not have a mental representation (Friedrich et al., 2006). The non-priming studies show that this difference cannot be found in infants yet up until at least 20 months of age. On the one hand, this might indicate that infants process the semantic meaning of a word when it is presented with a semantic context (e.g., a picture) but are not sensitive to the difference between real and pseudowords yet. That is, as pseudowords are words that sound like they belong to the target language but do not actually exist in that language, for infants almost all words they hear in their daily life are pseudowords to them. Not much is known yet about how this type of N400 effect develops. Future studies are encouraged to replicate these kinds of experiments with older children from age 2 years onwards.

Moreover, as we did not systematically compare studies that used the same paradigms but reported different ERP components, our systematic review does not allow us to understand how immature ERP components precede the presence of the N400. For the semantic congruity paradigms, which is the dominant paradigm to elicit linguistic N400 in infants, research with younger infants (e.g., 12-month-olds in Friedrich and Friederici, 2005a) showed that while infants were sensitive to congruent vs. incongruent pairings, this was visible in the N200–500, but not in the infant-like N400. Some studied showed that the N400 and N200–500 can co-exist, as they have different distributions (in 14-month-olds, Friedrich and Friederici, 2005a). Other studies also report the N200–500 as an index of novel word learning in a picture context (Friedrich and Friederici, 2008, 2011, 2017; Torkildsen et al., 2008). Indeed, the N200–500 priming and repetition effects have been found in infants as young as 3 months old (Friedrich and Friederici, 2017).

Finally, we calculated development of the N400 using pre-defined pre-processing steps, recording montages, and researchers' choice of time windows and distribution factors. There are various researchers' degrees of freedom in selecting ways to analyze the data, where researchers often follow lab traditions (Paul et al., 2021). Consequently, the results from our systematic review could be biased, as studies did not (nor were they designed to) agree on paradigms, recording settings, pre-processing steps, and quantification of the N400, all of which could affect the physical characteristics of the N400.

### Future Studies and Recommendations

The large heterogeneity in both latency and distribution shown to be present across studies highlight the need for more consistent methodological approaches across studies to fully characterize whether there are any developmental changes in the N400 effect. One welcome addition to the field would be cross-sectional or ideally even longitudinal studies with the same paradigm using small consecutive age-bins, and systematically testing on- and offsets of the N400 latency, together with reports on peak latency. Yet even then infant data might be contaminated with too much noise to infer development (van der Velde and Junge, 2020). Large data sets, such as acquired in a large-scale replication study as is recently published with adults and EEG on semantic processing, allow for more fine-grained analyses (Nieuwland et al., 2020). Possibly this could be achieved *via* a multi-lab collaborative effort focusing on replication at a larger scale to improve our understanding of infant methodology (Frank et al., 2017a). Recently, such an effort has been initiated with the ManyBabies consortium, who in their first replication project included more than 60 labs world-wide that all tested infants in behavioral experimental studies on their preference for infant-directed speech over adult-directed speech (ManyBabies Consortium, 2020). To our knowledge, a similar project but with EEG and a semantic congruity paradigm has not yet been proposed. Such large-scale replications could then also systematically investigate other task parameters, such as choices in design (for example proportion congruous: incongruous trials), timing intervals, and even other paradigms than the intermodal visual object-auditory word priming paradigms.

Moreover, in order to verify or rule out age related changes in N400 latencies and distributions within the first 2 years of life, it would also be helpful for the field to adopt a consensus on best practices for how to measure the N400. For adults, guidelines have been suggested to improve reproducibility and consistency in EEG recordings (Keil et al., 2014). For EEG studies it has also been recommended that prior to data collection, researchers preregister their study, in which they outline their hypotheses and proposed analyses plan (Paul et al., 2021). A recent set of research standards particularly focused at the N400 component, but this was again aimed at adults, not infants (Šoškić et al., 2020). We believe that additional analyses in research using the same approach across studies and age groups is needed to characterize N400 latency effects. Here we propose that researchers should also include a characterization of the onset and offset of the N400 latency; for example, by measuring the onset and duration of significant effects in overlapping 50–100 ms epochs (see Kooijman et al., 2005). Alternatively, the Hoormann Window Analysis (Hoormann et al., 1998) could also be applied to examine N400 effect time windows; these analyses provide more information on the interaction between sample time and condition, both of which are considered as additional within-subject factors in the ANOVA. This technique has been applied to research with the action N400 effect in infants (see, for example, Reid et al., 2009; Michel et al., 2017; Langeloh et al., 2020). Another way could be determining the presence or absence of significant amplitude differences in specific time windows, e.g., 0–200, 200–400, 400–600, etc., but include at least a 400–600 ms time window (the time window most commonly reported). It would also be helpful to include a standard set of electrode sites in N400 analyses to compare across studies. For instance, report results from omnibus ANOVAs including more electrodes than just the centro-parietal ones. This would not rule out also analyzing additional sites if high density recordings were obtained. Studies could for instance report this “standard set of analyses” in supplementary analyses.

Another consideration for future studies to make is how valid it is to compare infants who differ in vocabulary size from same-age peers, for example high vs. low producers, on the same set of stimuli (Peter et al., 2019). Will this set of stimuli be equally familiar to both groups of infants? Imagine that there is a difference in the N400, with the N400 being smaller in the low producer group. This could suggest that semantic processing is not as mature as in their more proficient same-aged peers. Alternatively, another explanation could be that the lower producers understood fewer words from the stimuli array. It is therefore essential to tailor the experiment to a child's knowledge, and make sure that infants know all the words in the test. If children with small vocabularies can be shown to understand all the words in the stimuli, but still show a smaller N400, it might indicate different processing strategies or strength of the semantic integration. However, very few studies examine comprehension of the individual words used.

### Conclusion

To this end, we have systematically reviewed 30 publication records on lexical-semantic processing in infancy to examine whether there is development in the physical characteristics of the infant N400. We observed few consistent patterns, be it from the perspective of age, experimental designs, or other individual characteristics. This also makes it difficult to determine the functionality of the N400 component in the first 2 years of life. The current review focused on the case of testing vocabulary (that is, semantic integration) and word learning. We reviewed studies that presented N400 evidence both for existing words and for recently acquired words. While familiar words may already be integrated into semantic networks, word learning in general is said to be largely associative in development (cf. McMurray et al., 2012; Sloutsky et al., 2017). Thus, the N400 effect in children under the age of two, in this sense, could be a functional result of either semantic integration or spreading activation. With only 35 infant samples included in this systematic review and the results of said studies varying to the degree we have found, preemptively declaring a functional interpretation would only be conjecture. A more systematic overview of the N400 effect in the first 2 years of life would be necessary in order to determine this and to understand the variability in the effect as a result of paradigm, age, and individual differences.

## Data Availability Statement

The original contributions generated for the study are included in the article/Supplementary Materials, further inquiries can be directed to the corresponding author.

## Author Contributions

CJ conceived of the idea and supervised the project. MB initiated the systematic search and entered results. CJ, MP, and SC verified results. All authors conferred in cases of doubt, provided critical feedback, and helped shape the research, analysis, and manuscript. CJ and MB carried out analyses and created figures. CJ, DM, and SC took the lead in writing the manuscript.

## Conflict of Interest

The authors declare that the research was conducted in the absence of any commercial or financial relationships that could be construed as a potential conflict of interest.

## Footnotes

^1^For one record (Torkildsen et al., 2008) we chose to report within-subject N400 findings separately for the existing word and pseudoword conditions, as these conditions were not statistically contrasted. Thus we had 35 participant samples, which yielded 36 data points for some analyses (e.g., choice of paradigm).

## References

[B1] Arias-Trejo N. Plunkett K. (2013). What's in a link: associative and taxonomic priming effects in the infant lexicon. Cognition 128, 214–227. 10.1016/j.cognition.2013.03.008 23688648

[B2] Asano M. Imai M. Kita S. Kitajo K. Okada H. Thierry G. (2015). Sound symbolism scaffolds language development in preverbal infants. Cortex 63, 196–205. 10.1016/j.cortex.2014.08.025 25282057

[B3] Aslin R. N. (2007). What's in a look? Dev. Sci. 10, 48–53. 10.1111/j.1467-7687.2007.00563.x 17181699 PMC2493049

[B4] Atchley R. A. Rice M. L. Betz S. K. Kwasny K. M. Sereno J. A. Jongman A. (2006). A comparison of semantic and syntactic event related potentials generated by children and adults. Brain Lang. 99, 236–246. 10.1016/j.bandl.2005.08.005 16226804

[B5] Azhari A. Truzzi A. Neoh M. J.-Y. Balagtas J. P. M. Tan H. H. Goh P. P.. (2020). A decade of infant neuroimaging research: what have we learned and where are we going? Infant Behav. Dev. 58:101389. 10.1016/j.infbeh.2019.101389 31778859

[B6] Baggio G. Hagoort P. (2011). The balance between memory and unification in semantics: a dynamic account of the N400. Lang. Cogn. Processes 26, 1338–1367. 10.1080/01690965.2010.542671

[B7] Barrett S. E. Rugg M. D. (1990). Event-related potentials and the semantic matching of pictures. Brain Cogn. 14, 201–212. 10.1016/0278-2626(90)90029-N 2285513

[B8] Bell M. A. Cuevas K. (2012). Using EEG to study cognitive development: issues and practices. J. Cogn. Dev. 13, 281–294. 10.1080/15248372.2012.691143 23144592 PMC3491357

[B9] Bentin S. (1987). Event-related potentials, semantic processes, and expectancy factors in word recognition. Brain Lang. 31, 308–327. 10.1016/0093-934X(87)90077-0 3620905

[B10] Bentin S. Allison T. Puce A. Perez E. McCarthy G. (1996). Electrophysiological studies of face perception in humans. J. Cognit. Neurosci. 8, 551–565. 10.1162/jocn.1996.8.6.551 20740065 PMC2927138

[B11] Bergelson E. Swingley D. (2012). At 6–9 months, human infants know the meanings of many common nouns. Proc. Natl. Acad. Sci. U.S.A. 109, 3253–3258. 10.1073/pnas.1113380109 22331874 PMC3295309

[B12] Bergmann C. Tsuji S. Piccinini P. E. Lewis M. L. Braginsky M. Frank M. C. Cristia A. (2018). Promoting replicability in developmental research through metaanalyses: Insights from language acquisition research. Child Dev. 89, 1996–2009. 10.1111/cdev.13079 29736962 PMC6282795

[B13] Borgström K. Torkildsen J. V. K. Lindgren M. (2015a). Event-related potentials during word mapping to object shape predict toddlers' vocabulary size. Front. Psychol. 6:143. 10.3389/fpsyg.2015.00143 25762957 PMC4327527

[B14] Borgström K. Torkildsen J. v. K. Lindgren M. (2015b). Substantial gains in word learning ability between 20 and 24 months: a longitudinal ERP study. Brain Lang. 149, 33–45. 10.1016/j.bandl.2015.07.002 26185047

[B15] Cantiani C. Riva V. Piazza C. Melesi G. Mornati G. Bettoni R.. (2017). ERP responses to lexical-semantic processing in typically developing toddlers, in adults, and in toddlers at risk for language and learning impairment. Neuropsychologia 103, 115–130. 10.1016/j.neuropsychologia.2017.06.031 28669897

[B16] Cosper S. H. (2020). The perceptual basis of meaning acquisition: Auditory associative word learning and the effect of object modality on word learning in infancy and adulthood (Doctoral Dissertation). Universität Osnabrück. Available online at: https://repositorium.ub.uni-osnabrueck.de/handle/urn:nbn:de:gbv:700-202011193766

[B17] Cosper S. H. Männel C. Mueller J. L. (2020). In the absence of visual input: electrophysiological evidence of infants' mapping of labels onto auditory objects. Dev. Cogn. Neurosci. 45:100821. 10.1016/j.dcn.2020.100821 32658761 PMC7358178

[B18] De Haan M. (2007). Infant EEG and Event-Related Potentials. Hove: Psychology Press.

[B19] DeBoer T. Scott L. S. Nelson C. A. (2007). Methods for acquiring and analyzing infant event-related potentials. Infant EEG Event Related Potentials 500, 5–37. 10.4324/9780203759660

[B20] Desroches A. S. Newman R. L. Joanisse M. F. (2009). Investigating the time course of spoken word recognition: electrophysiological evidence for the influences of phonological similarity. J. Cogn. Neurosci. 21, 1893–1906. 10.1162/jocn.2008.21142 18855555 PMC3965566

[B21] Di Lorenzo R. van den Boomen C. Kemner C. Junge C. (2020). Charting development of ERP components on face-categorization: results from a large longitudinal sample of infants. Dev. Cogn. Neurosci. 45:100840. 10.1016/j.dcn.2020.100840 32877890 PMC7476229

[B22] Duta M. D. Styles S. J. Plunkett K. (2012). ERP correlates of unexpected word forms in a picture–word study of infants and adults. Dev. Cogn. Neurosci. 2, 223–234. 10.1016/j.dcn.2012.01.003 22483072 PMC3336206

[B23] Eimer M. (2011). The face-sensitive N170 component of the event-related brain potential. Oxford Handbook Face Percept. 28, 329–344. 10.1093/oxfordhb/9780199559053.013.0017

[B24] Fenson L. Dale P. S. Reznick J. S. Bates E. Thal D. J. Pethick S. J.. (1994). Variability in early communicative development. Monogr. Soc. Res. Child Dev. 59:185. 10.2307/1166093 7845413

[B25] Ference J. Curtin S. (2015). The ability to map differentially stressed labels to objects predicts language development at 24 months in 12-month-olds at high risk for autism. Infancy 20, 242–262. 10.1111/infa.12074

[B26] Fernald A. Marchman V. A. (2012). Individual differences in lexical processing at 18 months predict vocabulary growth in typically developing and late-talking toddlers. Child Dev. 83, 203–222. 10.1111/j.1467-8624.2011.01692.x 22172209 PMC3266972

[B27] Fernald A. Perfors A. Marchman V. A. (2006). Picking up speed in understanding: speech processing efficiency and vocabulary growth across the 2nd year. Dev. Psychol. 42:98. 10.1037/0012-1649.42.1.98 16420121 PMC3214591

[B28] Fernald A. Pinto J. P. Swingley D. Weinberg A. McRoberts G. W. (1998). Rapid gains in speed of verbal processing by infants in the 2nd year. Psychol. Sci. 9, 228–231. 10.1111/1467-9280.00044

[B29] Forgács B. Gervain J. Parise E. Csibra G. Gergely G. Baross J.. (2020). Electrophysiological investigation of infants' understanding of understanding. Dev. Cogn. Neurosci. 43, 1–8. 10.1016/j.dcn.2020.100783 32510346 PMC7218257

[B30] Forgács B. Parise E. Csibra G. Gergely G. Jacquey L. Gervain J. (2019). Fourteen-month-old infants track the language comprehension of communicative partners. Dev. Sci. 22:e12751. 10.1111/desc.12751 30184313 PMC6492012

[B31] Frank M. C. Bergelson E. Bergmann C. Cristia A. Floccia C. Gervain J.. (2017a). A collaborative approach to infant research: promoting reproducibility, best practices, and theory-building. Infancy 22, 421–435. 10.1111/infa.12182 31772509 PMC6879177

[B32] Frank M. C. Braginsky M. Yurovsky D. Marchman V. A. (2017b). Wordbank: an open repository for developmental vocabulary data. J. Child Lang. 44:677. 10.1017/S0305000916000209 27189114

[B33] Friedrich C. K. Eulitz C. Lahiri A. (2006). Not every pseudoword disrupts word recognition: an ERP study. Behav. Brain Funct. 2:36. 10.1186/1744-9081-2-36 17062152 PMC1635431

[B34] Friedrich M. Friederici A. D. (2004). N400-like semantic incongruity effect in 19-month-olds: processing known words in picture contexts. J. Cogn. Neurosci. 16, 1465–1477. 10.1162/0898929042304705 15509391

[B35] Friedrich M. Friederici A. D. (2005a). Phonotactic knowledge and lexical-semantic processing in one-year-olds: brain responses to words and nonsense words in picture contexts. J. Cogn. Neurosci. 17, 1785–1802. 10.1162/089892905774589172 16269114

[B36] Friedrich M. Friederici A. D. (2005b). Semantic sentence processing reflected in the event-related potentials of one- and two-year-old children. Neuroreport 16, 1801–1804. 10.1097/01.wnr.0000185013.98821.62 16237330

[B37] Friedrich M. Friederici A. D. (2006). Early N400 development and later language acquisition. Psychophysiology 43, 1–12. 10.1111/j.1469-8986.2006.00381.x 16629680

[B38] Friedrich M. Friederici A. D. (2008). Neurophysiological correlates of online word learning in 14-month-old infants. Neuroreport 19, 1757–1761. 10.1097/WNR.0b013e328318f014 18955904

[B39] Friedrich M. Friederici A. D. (2010). Maturing brain mechanisms and developing behavioral language skills. Brian lang. 114, 66–71. 10.1016/j.bandl.2009.07.004 19665783

[B40] Friedrich M. Friederici A. D. (2011). Word learning in 6-month-olds: fast encoding-weak retention. J. Cogn. Neurosci. 23, 3228–3240. 10.1162/jocn_a_00002 21391764

[B41] Friedrich M. Friederici A. D. (2017). The origins of word learning: brain responses of 3-month-olds indicate their rapid association of objects and words. Dev. Sci. 20, 1–13. 10.1111/desc.12357 26548459

[B42] Friedrich M. Mölle M. Friederici A. D. Born J. (2019). The reciprocal relation between sleep and memory in infancy: memory- dependent adjustment of sleep spindles and spindle-dependent improvement of memories. Dev. Sci. 22:e12743. 10.1111/desc.12743 30160012 PMC6585722

[B43] Friedrich M. Mölle M. Friederici A. D. Born J. (2020). Sleep-dependent memory consolidation in infants protects new episodic memories from existing semantic memories. Nat. Commun. 11, 1–9. 10.1038/s41467-020-14850-8 32157080 PMC7064567

[B44] Friedrich M. Weber C. Friederici A. D. (2004). Electrophysiological evidence for delayed mismatch response in infants at-risk for specific language impairment. Psychophysiology 41, 772–782. 10.1111/j.1469-8986.2004.00202.x 15318883

[B45] Friedrich M. Wilhelm I. Born J. Friederici A. D. (2015). Generalization of word meanings during infant sleep. Nat. Commun. 6, 1–9. 10.1038/ncomms7004 25633407 PMC4316748

[B46] Friedrich M. Wilhelm I. Mölle M. Born J. Friederici A. D. (2017). The sleeping infant brain anticipates development. Curr. Biol. 27, 2374–2380. 10.1016/j.cub.2017.06.070 28756948

[B47] Gershkoff-Stowe L. (2002). Object naming, vocabulary growth, and the development of word retrieval abilities. J. Memory Lang. 46:665–687. 10.1006/jmla.2001.2830

[B48] Goldfield B. A. Reznick J. S. (1990). Early lexical acquisition: rate, content, and the vocabulary spurt. J. Child Lang. 17:171–183. 10.1017/S0305000900013167 2312640

[B49] Golinkoff R. M. Ma W. Song L. Hirsh-Pasek K. (2013). Twenty-five years using the intermodal preferential looking paradigm to study language acquisition: what have we learned? Perspect. Psychol. Sci. 8, 316–339. 10.1177/1745691613484936 26172975

[B50] Hagoort P. Baggio G. Willems R. M. (2009). Semantic unification, in The cognitive Neurosciences, 4th Edn, eds Gazzaniga M. S. (Cambridge, MA: MIT Press), 819–836.

[B51] Halit H. De Haan M. Johnson M. H. (2003). Cortical specialisation for face processing: face-sensitive event-related potential components in 3-and 12-month-old infants. Neuroimage 19, 1180–1193. 10.1016/S1053-8119(03)00076-4 12880843

[B52] Helo A. Azaiez N. Rama P. (2017). Word processing in scene context: an event-related potential study in young children. Dev. Neuropsychol. 42, 482–494. 10.1080/87565641.2017.1396604 29178812

[B53] Henderson L. M. Baseler H. A. Clarke P. J. Watson S. Snowling M. J. (2011). The N400 effect in children: relationships with comprehension, vocabulary and decoding. Brain Lang. 117, 88–99. 10.1016/j.bandl.2010.12.003 21272930

[B54] Hendrickson K. Love T. Walenski M. Friend M. (2019). The organization of words and environmental sounds in the second year: behavioral and electrophysiological evidence. Dev. Sci. 22:e12746. 10.1111/desc.12746 30159958 PMC6294716

[B55] Hirotani M. Stets M. Striano T. Friederici A. D. (2009). Joint attention helps infants learn new words: event-related potential evidence. Neuroreport 20, 600–605. 10.1097/WNR.0b013e32832a0a7c 19287321

[B56] Holcomb P. J. (1988). Automatic and attentional processing: an event-related brain potential analysis of semantic priming. Brain Lang. 35, 66–85. 10.1016/0093-934X(88)90101-0 3179703

[B57] Holcomb P. J. Coffey S. A. Neville H. J. (1992). Visual and auditory sentence processing: a developmental analysis using event-related brain potentials. Dev. Neuropsychol. 8, 203–241. 10.1080/87565649209540525

[B58] Hoormann J. Falkenstein M. Schwarzenau P. Hohnsbein J. (1998). Methods for the quantification and statistical testing of ERP differences across conditions. Behav. Res. Methods Instruments Comput. 30, 103–109. 10.3758/BF03209420

[B59] Iacoboni M. (2005). Understanding others: imitation, language, and empathy, in Perspectives on Imitation: From Neuroscience to Social Science: Vol. 1. Mechanisms of Imitation and Imitation in Animals, eds Hurley S. Chater N. (Cambridge, MA: MIT Press), 77–99.

[B60] Joyal M. Grouleau C. Bouchard C. Wilson M. A. Fecteau S. (2020). Semantic processing in healthy aging and Alzheimer's disease: a systematic review of the N400 differences. Brain Sci. 10:770. 10.3390/brainsci10110770 33114051 PMC7690742

[B61] Junge C. Cutler A. Hagoort P. (2012a). Electrophysiological evidence of early word learning. Neuropsychologia 50, 3702–3712. 10.1016/j.neuropsychologia.2012.10.012 23108241

[B62] Junge C. Everaert E. Porto L. Fikkert P. de Klerk M. Keij B.. (2020). Contrasting behavioral looking procedures: a case study on infant speech segmentation. Infant Behav. Dev. 60:101448. 10.1016/j.infbeh.2020.101448 32593957

[B63] Junge C. Kooijman V. Hagoort P. Cutler A. (2012b). Rapid recognition at 10 months as a predictor of language development. Dev. Sci. 15, 463–473. 10.1111/j.1467-7687.2012.1144.x 22709396

[B64] Kaduk K. Bakker M. Juvrud J. Gredebäck G. Westermann G. Lunn J.. (2016). Semantic processing of actions at 9 months is linked to language proficiency at 9 and 18 months. Int. J. Exp. Child Psychol. 151, 96–108. 10.1016/j.jecp.2016.02.003 26971305

[B65] Keil A. Debener S. Gratton G. Junghöfer M. Kappenman E. S. Luck S. J.. (2014). Committee report: publication guidelines and recommendations for studies using electroencephalography and magnetoencephalography. Psychophysiology 51, 1–21. 10.1111/psyp.12147 24147581

[B66] Kidd E. Donnelly S. (2020). Individual differences in first language acquisition. Annu. Rev. Linguist. 6, 319–340. 10.1146/annurev-linguistics-011619-030326

[B67] Kiefer M. (2002). The N400 is modulated by unconsciously perceived masked words: further evidence for an automatic spreading activation account of N400 priming effects. Cogn. Brain Res. 13, 27–39. 10.1016/S0926-6410(01)00085-4 11867248

[B68] Kooijman V. Hagoort P. Cutler A. (2005). Electrophysiological evidence for prelinguistic infants' word recognition in continuous speech. Cogn. Brain Res. 24, 109–116. 10.1016/j.cogbrainres.2004.12.009 15922163

[B69] Kutas M. Federmeier K. D. (2011). Thirty years and counting: finding meaning in the N400 component of the event-related brain potential (ERP). Annu. Rev. Psychol. 62, 621–647. 10.1146/annurev.psych.093008.131123 20809790 PMC4052444

[B70] Kutas M. Hillyard S. A. (1980). Reading senseless sentences: brain potentials reflect semantic incongruity. Science 207, 203–205. 10.1126/science.7350657 7350657

[B71] Langeloh M. Buttelmann D. Pauen S. Hoehl S. (2020). 12-to 14-month-olds expect unconstrained agents to act efficiently: event-related potential (ERP) evidence from the head-touch paradigm. Dev. Psychol. 56:1252. 10.1037/dev0000934 32324015

[B72] Lau E. F. Phillips C. Poeppel D. (2008). A cortical network for semantics:(de) constructing the N400. Int. Nat. Rev. Neurosci. 9, 920–933. 10.1038/nrn2532 19020511

[B73] Lazenby D. C. Sideridis G. D. Huntington N. Prante M. Dale P. S. Curtin S.. (2016). Language differences at 12 months in infants who develop autism spectrum disorder. J. Autism Dev. Disord. 46, 899–909. 10.1007/s10803-015-2632-1 26476738 PMC5497684

[B74] Leppänen P. H. Guttorm T. K. Pihko E. Takkinen S. Eklund K. M. Lyytinen H. (2004). Maturational effects on newborn ERPs measured in the mismatch negativity paradigm. Exp. Neurol. 190, 91–101. 10.1016/j.expneurol.2004.06.002 15498547

[B75] Mani N. Mills D. L. Plunkett K. (2012). Vowels in early words: an event-related potential study. Dev. Sci. 15, 2–11. 10.1111/j.1467-7687.2011.01092.x 22251287

[B76] ManyBabies Consortium (2020). Quantifying sources of variability in infancy research using the infant-directed-speech preference. Adv. Methods Prac. Psychol. Sci. 3, 24–52. 10.1177/2515245919900809

[B77] McMurray B. Horst J. S. Samuelson L. K. (2012). Word learning emerges from the interaction of online referent selection and slow associative learning. Psychol. Rev. 119:831. 10.1037/a0029872 23088341 PMC3632668

[B78] Michel C. Kaduk K. Ni Choisdealbha Á. Reid V. M. (2017). Event-related potentials discriminate familiar and unusual goal outcomes in 5-month-olds and adults. Dev. Psychol. 53:1833. 10.1037/dev0000376 28805436 PMC5611762

[B79] Mills D. L. Coffey-Corina S. Neville H. J. (1997). Language comprehension and cerebral specialization from 13 to 20 months. Dev. Neuropsychol. 13, 397–445. 10.1080/87565649709540685

[B80] Mills D. L. Coffey-Corina S. A. Neville H. J. (1993). Language-acquisition and cerebral specialization in 20-month-old infants. J. Cogn. Neurosci. 5, 317–334. 10.1162/jocn.1993.5.3.317 23972220

[B81] Mills D. L. Conboy B. Paton C. (2005a). Do changes in brain organization reflect shifts in symbolic functioning?, in Symbol Use and Symbolic Representation, ed Namy L. L. (New York, NY: Erlbaum), 123–153.

[B82] Mills D. L. Plunkett K. Prat C. Schafer G. (2005b). Watching the infant brain learn words: effects of vocabulary size and experience. Cogn. Dev. 20, 19–31. 10.1016/j.cogdev.2004.07.001

[B83] Mills D. L. Prat C. Zangl R. Stager C. L. Neville H. J. Werker J. F. (2004). Language experience and the organization of brain activity to phonetically similar words: ERP evidence from 14-and 20-month-olds. J. Cogn. Neurosci. 16, 1452–1464. 10.1162/0898929042304697 15509390

[B84] Moher D. Liberati A. Tetzlaff J. Altman D. G. Group P. (2009). Preferred reporting items for systematic reviews and meta-analyses: the PRISMA statement. PLoS Med. 6:e1000097. 10.1371/journal.pmed.1000097 19621072 PMC2707599

[B85] Morgan E. U. der Meer A. V. Vulchanova M. Blasi D. E. Baggio G. (2020). Meaning before grammar: a review of ERP experiments on the neurodevelopmental origins of semantic processing. Psychonomic Bull. Rev. 27, 1–24. 10.3758/s13423-019-01677-8 31950458

[B86] Näätänen R. (1990). The role of attention in auditory information processing as revealed by event-related potentials and other brain measures of cognitive function. Behav. Brain Sci. 13, 201–233. 10.1017/S0140525X00078407

[B87] Nazzi T. Bertoncini J. (2003). Before and after the vocabulary spurt: two modes of word acquisition? Dev. Sci. 6, 136–142. 10.1111/1467-7687.00263

[B88] Newman R. L. Connolly J. F. (2009). Electrophysiological markers of pre-lexical speech processing: evidence for bottom–up and top–down effects on spoken word processing. Biol. Psychol. 80, 114–121. 10.1016/j.biopsycho.2008.04.008 18524453

[B89] Nieuwland M. S. Barr D. J. Bartolozzi F. Busch-Moreno S. Darley E. Donaldson D. I.. (2020). Dissociable effects of prediction and integration during language comprehension: evidence from a large-scale study using brain potentials. Philos. Trans. R. Soc. B Biol. Sci. 375:20180522. 10.1098/rstb.2018.0522 31840593 PMC6939355

[B90] Obrig H. Mock J. Stephan F. Richter M. Vignotto M. Rossi S. (2017). Impact of associative word learning on phonotactic processing in 6-month- old infants: a combined EEG and fNIRS study. Dev. Cogn. Neurosci. 25, 185–197. 10.1016/j.dcn.2016.09.001 27692617 PMC6987754

[B91] Parise E. Csibra G. (2012). Electrophysiological evidence for the understanding of maternal speech by 9-month-old infants. Psychol. Sci. 23, 728–733. 10.1177/0956797612438734 22692337 PMC4641316

[B92] Parise E. Handl A. Palumbo L. Friederici A. D. (2011). Influence of eye gaze on spoken word processing: an ERP study with infants. Child Dev. 82, 842–853. 10.1111/j.1467-8624.2010.01573.x 21410929

[B93] Pasman J. W. Rotteveel J. J. Maassen B. Visco Y. M. (1999). The maturation of auditory cortical evoked responses between (preterm) birth and 14 years of age. Euro. J. Paediatric Neurol. 3, 79–82. 10.1016/S1090-3798(99)80017-8 10700543

[B94] Paul M. Govaart G. H. Schettino A. (2021). Making ERP research more transparent: guidelines for preregistration. Int. J. Psychophysiol. 164, 52–63. 10.1016/j.ijpsycho.2021.02.016 33676957

[B95] Peter M. S. Durrant S. Jessop A. Bidgood A. Pine J. M. Rowland C. F. (2019). Does speed of processing or vocabulary size predict later language growth in toddlers? Cogn. Psychol. 115:101238. 10.1016/j.cogpsych.2019.101238 31539813

[B96] Posner M. I. Snyder C. R. R. (1975). Attention and cognitive control, in Information Processing and Cognition: The Loyota Symposium, ed Solso R. L.(Erlbaum, Hillsdale, NJ), 55–85.

[B97] Pruden S. M. Hirsh-Pasek K. Golinkoff R. M. Hennon E. A. (2006). The birth of words: ten-month-olds learn words through perceptual salience. Child Dev. 77, 266–280. 10.1111/j.1467-8624.2006.00869.x 16611171 PMC4621011

[B98] Quine W. V. O. (1960). Word and Object: An Inquiry Into the Linguistic Mechanisms of Objective Reference. Cambridge, MA: MIT Press.

[B99] Rabovsky M. Hansen S. S. McClelland J. L. (2018). Modelling the N400 brain potential as change in a probabilistic representation of meaning. Nat. Human Behav. 2, 693–705. 10.1038/s41562-018-0406-4 31346278

[B100] Rämä P. Sirri L. Serres J. (2013). Development of lexical-semantic language system: N400 priming effect for spoken words in 18- and 24-month old children. Brain Lang. 125, 1–10. 10.1016/j.bandl.2013.01.009 23435193

[B101] Reid V. M. (2012). Introduction to the special issue: infant EEG comes of age. Dev. Neuropsychol. 37, 185–186. 10.1080/87565641.2012.668165 22545657

[B102] Reid V. M. Hoehl S. Grigutsch M. Groendahl A. Parise E. Striano T. (2009). The neural correlates of infant and adult goal prediction: evidence for semantic processing systems. Int. Dev. Psychol. 45, 620–629. 10.1037/a0015209 19413420

[B103] Rugg M. D. Curran T. (2007). Event-related potentials and recognition memory. Trends Cogn. Sci. 11, 251–257. 10.1016/j.tics.2007.04.004 17481940

[B104] Rugg M. D. Doyle M. C. Wells T. (1995). Word and nonword repetition within-and across-modality: an event-related potential study. J. Cogn. Neurosci. 7, 209–227. 10.1162/jocn.1995.7.2.209 23961825

[B105] Rugg M. D. Nagy M. E. (1987). Lexical contribution to nonword-repetition effects: evidence from event-related potentials. Memory Cogn. 15, 473–481. 10.3758/BF03198381 3695941

[B106] Sheehan E. A. Mills D. L. (2008). The effect of early word learning on brain development, in Early Language Development: Bridging Brain and Behaviour, eds Mills S. Friederici A. D. Thierry G. (Amsterdam; Philadelphia, PA: John Benjamins Publishing), 161–190.

[B107] Sheehan E. A. Namy L. L. Mills D. L. (2007). Developmental changes in neural activity to familiar words and gestures. Brain Lang. 101, 246–259. 10.1016/j.bandl.2006.11.008 17250885

[B108] Sirri L. Rämä P. (2015). Cognitive and neural mechanisms underlying semantic priming during language acquisition. J. Neurolinguist. 35, 1–12. 10.1016/j.jneuroling.2015.01.003

[B109] Sloutsky V. M. Yim H. Yao X. Dennis S. (2017). An associative account of the development of word learning. Cogn. Psychol. 97, 1–30. 10.1016/j.cogpsych.2017.06.001 28641208 PMC5666698

[B110] Smith L. Yu C. (2008). Infants rapidly learn word-referent mappings via cross-situational statistics. Cognition 106, 1558–1568. 10.1016/j.cognition.2007.06.010 17692305 PMC2271000

[B111] Šoškić A. Jovanoviæ V. Styles S. J. Kappenman E. S. Kovic V. (2020). How to do better N400 studies: reproducibility, consistency and adherence to research standards in the existing literature. PsyArXiv 10.31234/osf.io/jp6wy PMC9381463 34374003

[B112] Stets M. Stahl D. Reid V. M. (2012). A meta-analysis investigating factors underlying attrition rates in infant ERP studies. Dev. Neuropsychol. 37, 226–252. 10.1080/87565641.2012.654867 22545660

[B113] Swingley D. (2005). 11-month-olds' knowledge of how familiar words sound. Dev. Sci. 8, 432–443. 10.1111/j.1467-7687.2005.00432.x 16048516

[B114] Taylor M. J. Baldeweg T. (2002). Application of EEG, ERP and intracranial recordings to the investigation of cognitive functions in children. Dev. Sci. 5, 318–334. 10.1111/1467-7687.00372

[B115] Thierry G. (2005). The use of event-related potentials in the study of early cognitive development. Infant Child Dev. 14, 85–94. 10.1002/icd.353

[B116] Thornhill D. E. Van Petten C. (2012). Lexical versus conceptual anticipation during sentence processing: frontal positivity and N400 ERP components. Int. J. Psychophysiol. 83, 382–392. 10.1016/j.ijpsycho.2011.12.007 22226800

[B117] Tincoff R. Jusczyk P. W. (2012). Six-month-olds comprehend words that refer to parts of the body. Infancy 17, 432–444. 10.1111/j.1532-7078.2011.00084.x 32693484

[B118] Torkildsen J. V. K. Hansen H. F. Svangstu J. M. Smith L. Simonsen H. G. Moen I.. (2009). Brain dynamics of word familiarization in 20-month-olds: effects of productive vocabulary size. Brain Lang. 108, 73–88. 10.1016/j.bandl.2008.09.005 18950850

[B119] Torkildsen J. V. K. Sannerud T. Syversen G. Thormodsen R. Simonsen H. G. Moen I.. (2006). Semantic organization of basic-level words in 20-month-olds: an ERP study. J. Neurolinguist. 19, 431–454. 10.1016/j.jneuroling.2006.01.002

[B120] Torkildsen J. V. K. Svangstu J. M. Hansen H. F. Smith L. Simonsen H. G. Moen I.. (2008). Productive vocabulary size predicts event-related potential correlates of fast mapping in 20-month-olds. J. Cogn. Neurosci. 20, 1266–1282. 10.1162/jocn.2008.20087 18284350

[B121] Torkildsen J. V. K. Syversen G. Simonsen H. G. Moen I. Lindgren M. (2007a). Brain responses to lexical-semantic priming in children at-risk for dyslexia. Brain Lang. 102, 243–261. 10.1016/j.bandl.2006.11.010 17239944

[B122] Torkildsen J. V. K. Syversen G. Simonsen H. G. Moen I. Lindgren M. (2007b). Electrophysiological correlates of auditory semantic priming in 24-month-olds. J. Neurolinguist. 20, 332–351. 10.1016/j.jneuroling.2007.02.003

[B123] Travis K. E. Leonard M. K. Brown T. T. Hagler D. J. Jr. Curran M. Dale A. M.. (2011). Spatiotemporal neural dynamics of word understanding in 12- to 18-month- old-infants. Cereb. Cortex 21, 1832–1839. 10.1093/cercor/bhq259 21209121 PMC3138516

[B124] Van Berkum J. J. A. (2009). The neuropragmatics of 'simple' utterance comprehension: an ERP review, in Semantics and pragmatics: From Experiment to Theory, eds Sauerland U. Yatsushiro K. (Basingstoke: Palgrave Macmillan), 276–316.

[B125] van der Velde B. Junge C. (2020). Limiting data loss in infant EEG: putting hunches to the test. Dev. Cogn. Neurosci. 45:100809. 10.1016/j.dcn.2020.100809 32658760 PMC7358181

[B126] van Viersen S. de Bree E. H. Verdam M. Krikhaar E. Maassen B. van der Leij A.. (2017). Delayed early vocabulary development in children at family risk of dyslexia. J. Speech Lang. Hearing Res. 60, 937–949. 10.1044/2016_JSLHR-L-16-0031 28282655

[B127] Werker J. F. Hensch T. K. (2015). Critical periods in speech perception: new directions. Annu. Rev. Psychol. 66, 173–196. 10.1146/annurev-psych-010814-015104 25251488

[B128] Willems R. M. Özyürek A. Hagoort P. (2009). Differential roles for left inferior frontal and superior temporal cortex in multimodal integration of action and language. Neuroimage 47, 1992–2004. 10.1016/j.neuroimage.2009.05.066 19497376

[B129] Woodward A. L. Markman E. M. Fitzsimmons C. M. (1994). Rapid word learning in 13- and 18-month-olds. Dev. Psychol. 30, 553–566. 10.1037/0012-1649.30.4.553

