# Supplementary material for: Development of the N400 for Word Learning in the First 2 Years of Life: A Systematic Review
Source: Front Psychol. 2021 Jun 30;12:689534. doi: 10.3389/fpsyg.2021.689534 (PMC8277998; doi:10.3389/fpsyg.2021.689534)
Supplement: Supplementary file 1 [file Data_Sheet_1.docx]

**Appendix A**

Complete search strategies used in online databases PubMed, Google Scholar and Web of Science

1. PubMed

(semantic[Title/Abstract] OR word*[Title/Abstract] OR lexic*[Title/Abstract] OR senten*[Title/Abstract] OR vocabulary[Title/Abstract] OR speech[Title/Abstract] OR “Language Development”[MeSH Terms] OR Vocabulary[MeSH Terms] OR Semantics[MeSH Terms] OR “Speech Perception”[MeSH Terms])

AND

(infan*[Title/Abstract] OR toddler*[Title/Abstract] OR child[Title/Abstract] OR children[Title/Abstract] OR Infant[MeSH Terms])

AND

EEG[Title/Abstract] OR ERP[Title/Abstract] OR electrophysiolog*[Title/Abstract] OR N400[Title/Abstract] OR “event-related potential*”[Title/Abstract] OR “Brain Mapping”[MeSH Terms] OR Electroencephalography[MeSH Terms] OR “Evoked Potentials”[MeSH Terms]

1. Google Scholar

(semantic OR lexical)

AND

infant*

AND

N400

1. Web of Science

TOPIC: (semantic OR word* OR lexic* OR senten* OR vocabulary OR speech)

AND

TOPIC: (infan* OR toddler* OR child OR children)

AND

TOPIC: (EEG OR ERP OR electrophysiolog* OR N400 OR “event-related potential*”)
